# Early Pleistocene archaeological occurrences at the Feiliang site, and the archaeology of human origins in the Nihewan Basin, North China

**DOI:** 10.1371/journal.pone.0187251

**Published:** 2017-11-22

**Authors:** Shuwen Pei, Fei Xie, Chenglong Deng, Zhenxiu Jia, Xiaomin Wang, Ying Guan, Xiaoli Li, Dongdong Ma, Ignacio de la Torre

**Affiliations:** 1 Key Laboratory of Vertebrate Evolution and Human Origins, Institute of Vertebrate Paleontology and Paleoanthropology, Chinese Academy of Sciences, Beijing, China; 2 Hebei Provincial Institute of Cultural Relics, Shijiazhuang, China; 3 State Key Laboratory of Lithospheric Evolution, Institute of Geology and Geophysics, Chinese Academy of Sciences, Beijing, China; 4 University of Chinese Academy of Sciences, Beijing, China; 5 Beijing Museum of Natural History, Beijing, China; 6 Institute of Archaeology, University College London, London, United Kingdom; Seoul National University College of Medicine, REPUBLIC OF KOREA

## Abstract

The Early Pleistocene archaeological evidence from the fluvio-lacustrine sequence of the Nihewan Basin (North China) offers an excellent opportunity to explore early human evolution and behavior in a temperate setting in East Asia, following the earliest ‘Out of Africa’. Here we present the first comprehensive study of the Feiliang (FL) site, with emphasis on the archaeological sequence, site integrity, and stone artifact assemblages. Magnetostratigraphic dating results show that early humans occupied the site ca. 1.2 Ma. Archaeological deposits were buried rapidly in primary context within shallow lake margin deposits, with only minor post-depositional disturbance from relatively low energy hydraulic forces. The FL lithic assemblage is characterized by a core and flake, Oldowan-like or Mode 1 technology, with a low degree of standardization, expedient knapping techniques, and casually retouched flakes. The bone assemblage suggests that hominin occupation of the FL site was in an open habitat of temperate grassland with areas of steppe and water. The main features of the FL assemblage are discussed in the context of the early Pleistocene archaeology of Nihewan, for which an assessment of current and future research is also presented.

## Introduction

The earliest dispersal of hominins out of Africa constitutes a central issue in modern Paleoanthropology [1–3]. Current evidence of hominin fossils and stone artifacts indicate that sometime after 2 Ma (million years ago), hominins began to spread out of Africa, reaching Dmanisi (Georgia) by ~1.7 Ma [4, 5], and potentially eastern Asia by 1.8–1.6 Ma [6–13]. In addition to the probable southern route through Arabia and Southeast Asia [14–16], another plausible dispersal corridor could have included a northern route from Dmanisi through Mongolia, reaching the Nihewan Basin in northern China [9, 11, 17].

Previous and ongoing archaeological investigations in the Nihewan basin have yielded one of the world’s most important sequences for the study of the early Pleistocene [10, 11, 18–23], which offers an excellent opportunity to explore the archaeology of human origins in a temperate setting during the earliest time span of ‘Out of Africa I’[11, 24]. Nihewan early Pleistocene stone artifact assemblages have been described as technologically simple, and characterized by an apparently non-economical use of raw materials, generally informal artifacts and rare occurrence of retouched flakes [15, 25–28]. They have traditionally been attributed to an East Asian Oldowan-like / Mode 1 technology [29–31] rather than to the more advanced Acheulean technology (Mode 2)[32].

This paper will introduce the archaeological sequence of the Feiliang (hereafter FL) site complex, in the Nihewan basin, excavated in 1990 and 1996. We will focus on the archaeo-stratigraphic sequence, site formation processes and, particularly, on lithic technology and raw materials. Our aim is to present a detailed account of the Oldowan-like technology in the Early Pleistocene sequence of FL, and discuss the significance of well-preserved, low-density archaeological assemblages for the reconstruction of early Pleistocene hominin behavior in East Asia. In addition, we aim to discuss FL in the context of the early Pleistocene archaeology of the Nihewan basin.

## Materials and methods

### Ethics statement

1a. Specimen numbers: fossils and lithics from trench 1 were labelled with the site’s name (i.e. FL), while fossils and lithics from other trenches were labelled with the site’s name, trench, and the year they were excavated (i.e., 96FL-T2, 96FL-T3, 96FL-TOK), followed by a correlative number for each trench (e.g., FL-1, 96FL-TOK-1). A total of 3364 fossils and lithics received an accession number (i.e., FL-1∼564, 96FL-T2- 1∼644, 96FL-T3-1∼614, 96FL-TOK-1∼1542).

1b. All archaeological specimens reported in this paper are housed in the Hebei Provincial Institute of Cultural Relics, in Shijiazhuang, Hebei province, China. Access to these specimens is granted by the Hebei Provincial Institute of Cultural Relics.

1c. Permits were granted by the Basic Scientific Special Program of Ministry of Science and Technology of China (Grant No. 2014FY110300).

Field permit granted by the State Administration of Cultural Heritage, China.

### Geology and stratigraphy

**Fig 1:**
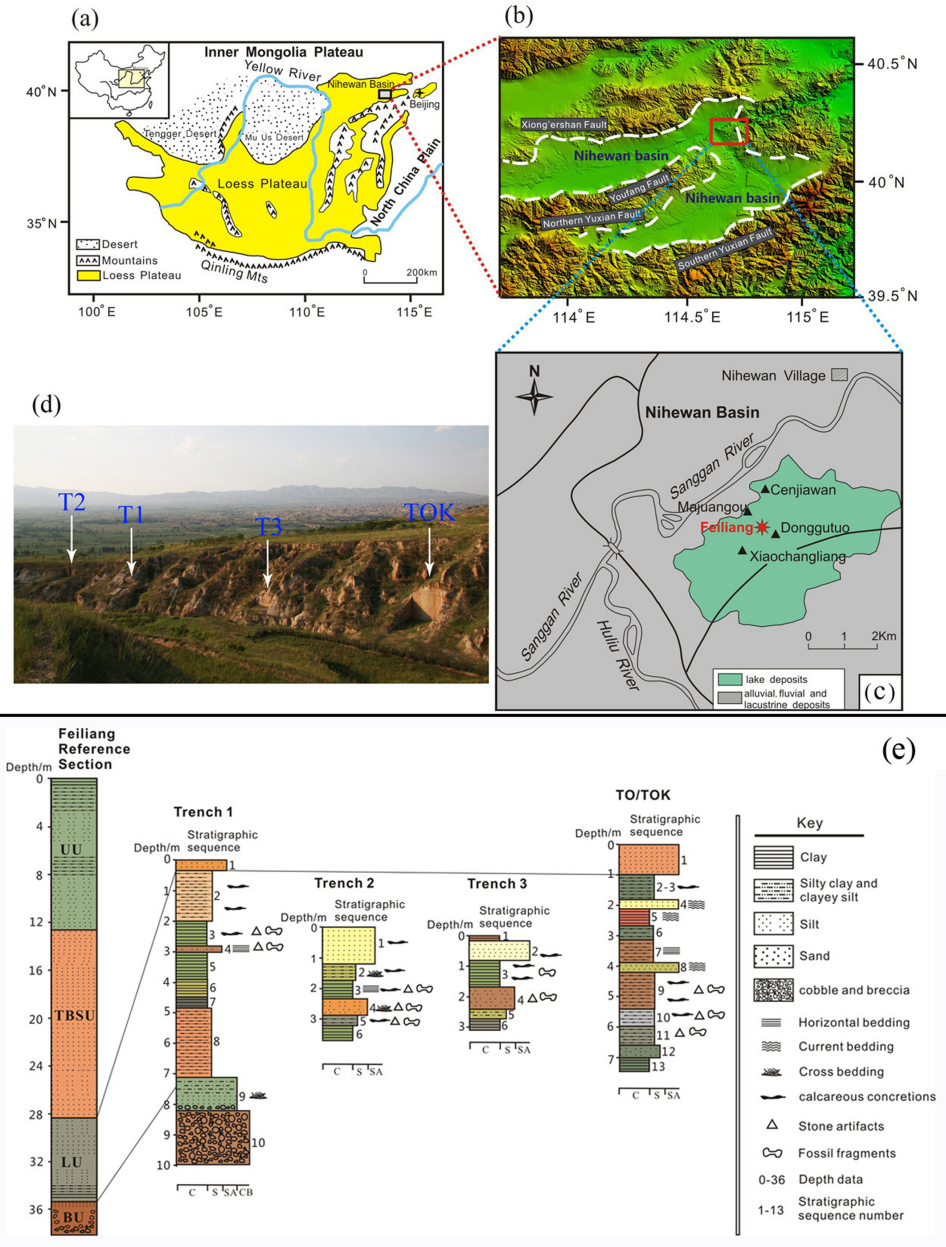
Schematic map showing the Chinese Loess Plateau, Nihewan basin, the main Early Paleolithic sites mentioned in the paper (modified from Zhu et al.[9], and Ao et al.[32]), and stratigraphic section of FL site. (a) Nihewan basin in North China; (b) Sketch map of the Nihewan basin; (c) relevant sites in the eastern part of the Nihewan basin; (d) View of the FL trenches (from the southeast). e) Composite sections of T1, T2, T3, and TOK, correlated with the Feiliang Type Section. BU-basal unit, LU-lower unit, TBSU-thick brown sand unit, UU-upper unit. C-clay, S-silt, SA-sand, CB-cobble and breccia.

The FL site complex is located in the Cenjiawan Platform on the eastern margin of the Nihewan basin, where several other important Early Pleistocene sites (e.g. Majuangou (MJG), Cenjiawan (CJW), Xiaochangliang (XCL), and Donggutuo (DGT) are located (Fig 1A–1C) (see also supporting information S1 Appendix). Here, the 38 m-thick exposed Nihewan fluvio-lacustrine deposits consist mainly of grayish-yellow and grayish-green silty clays, silts and sandy silts (Fig 1D). Fig 1E shows the stratigraphic units and position of the archaeological trenches in relation to the FL type section. The fluvio-lacustrine sequence lies on a distinctive unconformity, with several meters of topographic relief and over a lateral distance of several hundred meters. Four main sedimentological units are visible in the type section. The Basal Unit (BU), with a total thickness of 2.7 m, lies above the unconformity; it is formed of coarse-grained fluvial sediments (gravels), a brown yellow silty sand gravel layer with cobbles and pebbles, rounded clasts, and breccia. The base of the section is underlain by grey to red Jurassic volcanic breccia. Above the BU, the 6.9 m-thick Lower Unit (LU) consists predominantly of massive sandy silts, silt, and pale grey silty clay. This unit shows horizontal and ripple beddings, and contains calcareous nodules and concretions, ferruginous nodules and rust spots, and complete and fragmented mollusks. The next distinct unit is the Thick Brown Sand Unit (TBSU), with a thickness of 15.8 m and consisting of sand, silt, and clayey silt, all light brown in color. Thin horizontal lamination and ripple bedding are common. Above the TBSU, the Upper Unit (UP) extends for 12.6 m in the type section, and is formed of alternating light grey and light brown sand, silt, and clay. A dark gray clay that expands over 2 meters above the UP marks a well-developed weathering surface at the top of the Nihewan fluvio-lacustrine deposits. Loess sediments at the top of the section have been subjected to erosion, and are better preserved in some stratigraphic sections of a higher altitude than the FL sequence. Late Pleistocene tectonics and erosion shaped a northwest-southeast trending ridge of over 200 m in length tilted to the southwest. The FL archaeological trenches are placed in the lake-margin silts and clays at 26.2–26.7 m from the top, i.e. extending through sediments from the LU (Fig 1E).

### Dating

**Fig 2:**
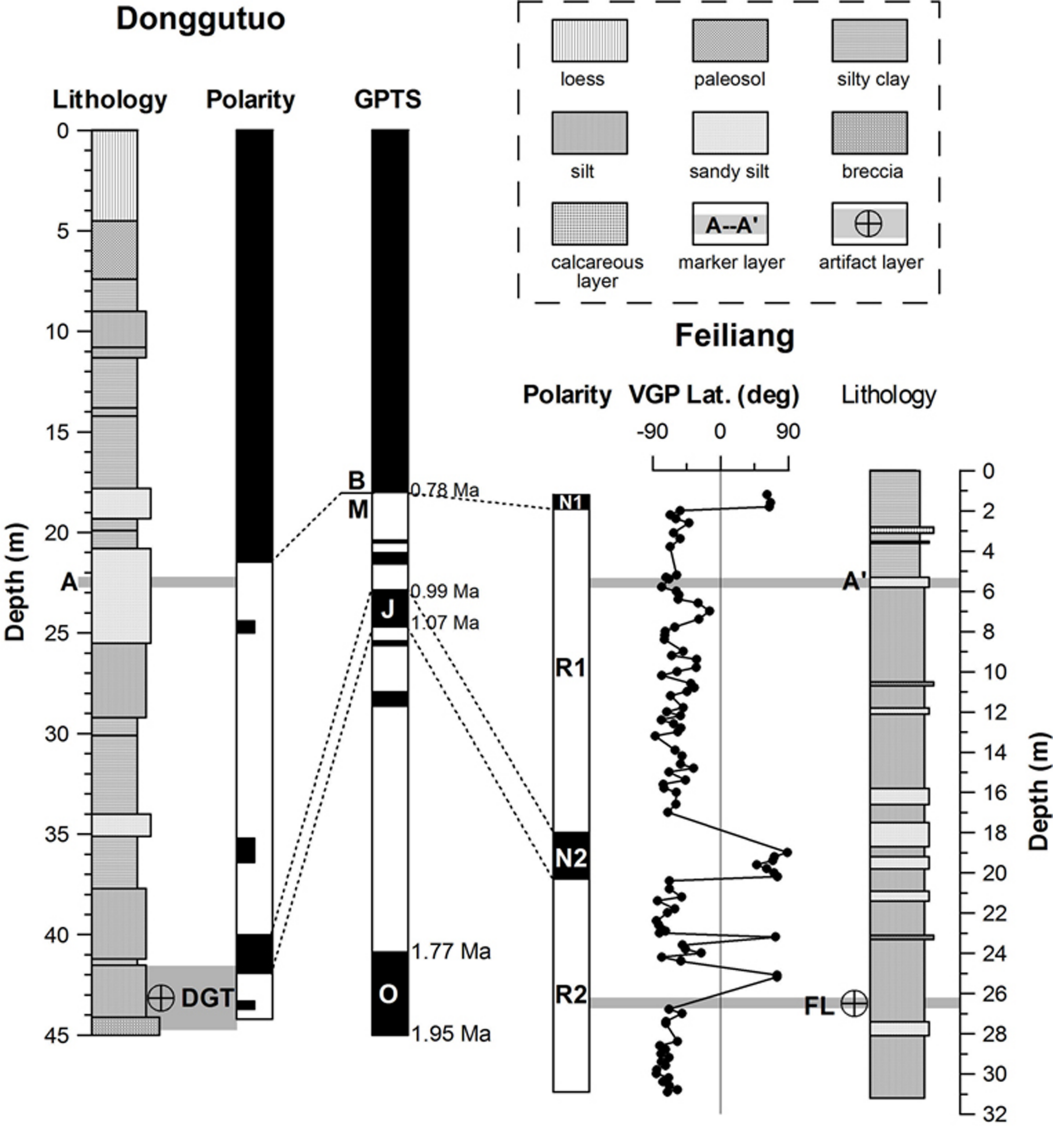
Lithostratigraphy and magnetic polarity stratigraphy of the Donggutuo (Wang et al.[34]) and Feiliang (Deng et al.[33]) sections, and their correlations with the geomagnetic polarity timescale (GPTS) (Cande and Kent [35]). B, Brunhes; M, Matuyama; O, Olduvai; J, Jaramillo; VGP Lat., latitude of the virtual geomagnetic pole. DGT and FL refer to the Donggutuo and Feiliang artifact layers, respectively.

Deng et al. [33] reported on the paleomagnetic stratigraphy and lithostratigraphy of artifact-bearing strata at the Feiliang site, spanning a thickness of 30.9 m (Fig 2) at Trench TOK (Fig 1E). The profiles of Trench TOK correspond to the section below the Jaramillo subchron in Fig 2, and the artifact layer, identified by Deng et al. [33] at the depth interval of 26.2–26.7 m (Fig 2), roughly corresponds to layer 11 of Trench TOK in Fig 1E.

Deng et al. [33] recognized four magnetozones: two normal, N1 (0–1.9 m) and N2 (18–20.3 m), and two reverse, R1 (1.9–18 m) and R2 (20.3–30.9 m). The stone artifact layer (at an average depth of 26.45 m) occurs within magnetozone R2 (Fig 2). A distinctive marker layer of yellow sandy silts is used for local stratigraphic correlation between the Feiliang and Donggutuo sections (Fig 2). On the basis of paleomagnetic and sedimentological data, magnetozones N1 and N2 are attributed respectively to the very early Brunhes chron and the Jaramillo subchron, and magnetozones R1 and R2 to the post- and pre-Jaramillo Matuyama chron, respectively (Fig 2). The age of the artifact layer was estimated by extrapolating the sedimentation rates of magnetozones R1-N2 (that is, between the Brunhes-Matuyama boundary and the lower boundary of the Jaramillo subchron) (Fig 2).

The fluvio-lacustrine Feiliang sedimentary sequence mainly comprises fine-grained sediments, including silty clays, silts and sandy silts, in which no disconformities are observed. Deng et al. [33] therefore estimate the age of the Feiliang artifact layer by extrapolating sediment accumulation rates (SARs). The average SARs of magnetozone N2 only (within the Jaramillo subchron) and magnetozones R1‒N2 (between Brunhes-Matuyama boundary and the lower boundary of the Jaramillo subchron) are 2.88 cm kyr^-1^ and 6.34 cm kyr^-1^, respectively; hence, the extrapolated age estimates for the Feiliang stone artifact layer are ca. 1.3 Ma and ca. 1.2 Ma, respectively [33]. Given the relatively high variability in SARs of the continental fluvio-lacustrine sequences, Deng et al. [33] consider ca. 1.2 Ma as the best approximation for the age of the Feiliang artifact layer (see also details in [34, 35]).

### Archaeological excavation

**Fig 3:**
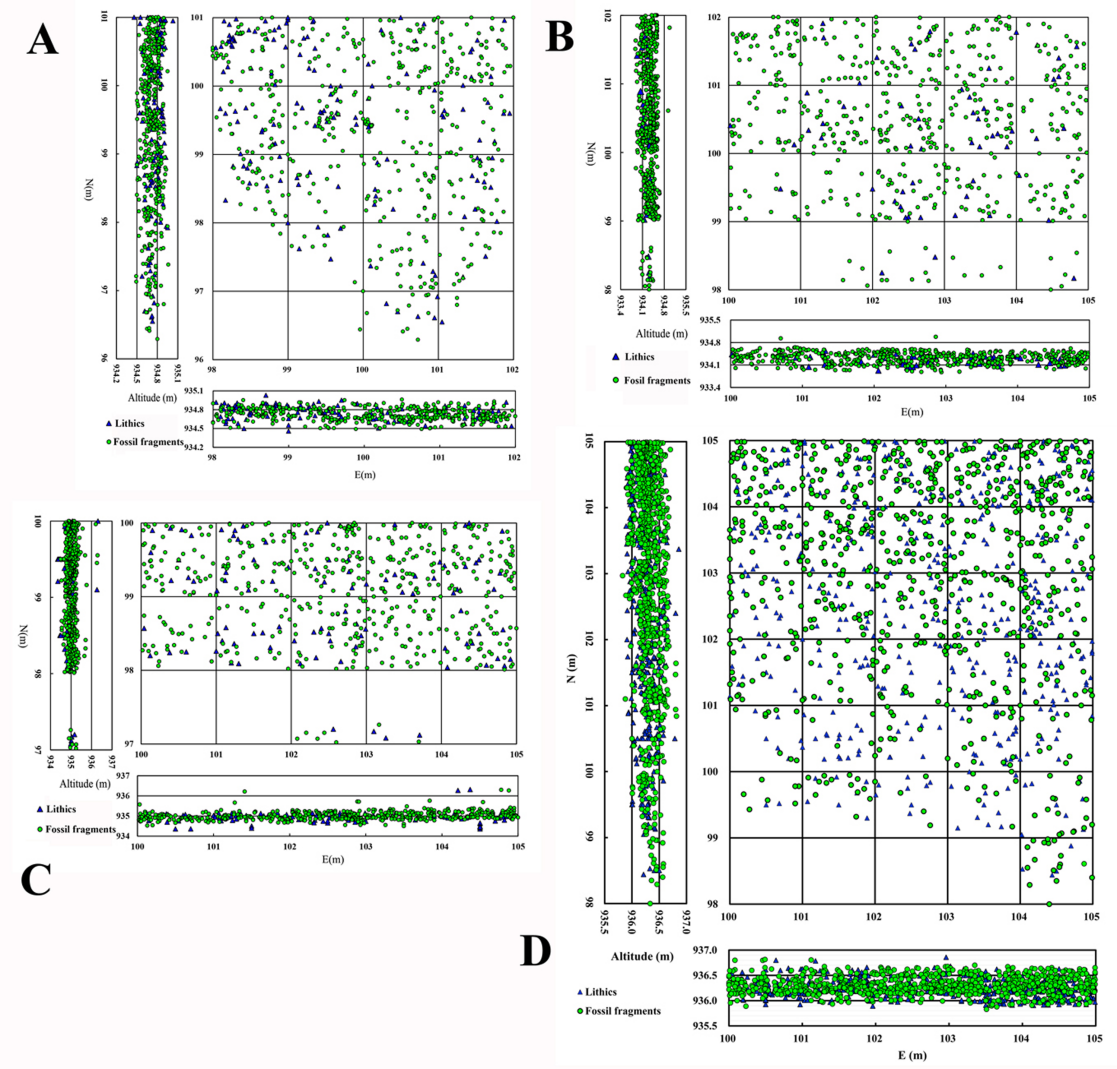
Horizontal and vertical distributions of excavated remains of the FL trenches. A) T1. B) T2. C) T3. D) TOK.

Feiliang (meaning small ridge in Chinese) was discovered in 1985 by one of us (FX), and was test-excavated in 1986. Trench 1 of the FL site was excavated in 1990, and Trench 2, Trench 3, and TOK in 1996 by a China- US international team. Systematic mapping and geomorphological study of the FL area was undertaken prior to excavations, focusing on the reference section for stratigraphic profiles of the fluvio-lacustrine deposits identified along the Feiliang ridge. All excavations were conducted in 2 to 5 cm spits, with larger spits used for sterile layers. Sediments were dry sieved with 5mm mesh. Horizontal and vertical distribution of excavated remains was recorded in all trenches, and shown in Fig 3. Preferred long axis orientations of lithic artifacts and bones have been recognized as a valuable means to assess water disturbance [36–38], and thus artifact orientation was measured with a compass during fieldwork.

Nearly 100 m^2^ were exposed, and 983 lithic artifacts and more than 2000 animal fossil fragments were collected (Table 1). The lithic assemblage from Trench 1 was reported by Xie et al. [39] and is fully re-analyzed in the present work, while the archaeological occurrences from Trench 2, Trench 3, and TOK will be reported here for the first time. A detailed description of the archaeo-stratigraphic and sedimentological features of each trench is available in supporting information S1 Table. The primary dataset used for this paper is available in supporting information S2 Table. Access to the FL archaeological materials can be requested to the Hebei Provincial Institute of Cultural Relics (Shijiazhuang, China).

**Table 1:** Main features of major trenches at the Feiliang site complex (FL). *Only lithics.

			Trench 1 (T1)	Trench 2 (T2)	Trench 3 (T3)	TOK
Year discovered			1985	1985	1985	1985
Year excavated			1990	1996	1996	1996
Location			N 40°13′27.2″ E 114°40′04.4″ +934.5–935.0m	N 40°13′27.5″ E 114°40′03.5″ +933.9–935.5m	N 40°13′26.3″ E 114°40′05.7″ +934.3–936.3m	N 40°13′26.0″ E 114°40′07.1″ +935.1–937.2m

Elevation (m.a.s.l/)						
Area excavated (m^2^)			17	18	12	35
Thickness of archaeological levels (cm)			50	160	195	210
Number of excavated spits			15	29	35	18
Archaeology	Surface*		22		4	2
	Excavated	lithics	111	77	92	669
		Fossils	431	567	518	871
Artifacts/m^2^			6.53	4.28	7.67	19.11

### Stone tool analysis

Fluvial abrasion of artifacts was evaluated using four indices: fresh, slightly abraded, abraded, and heavily abraded [40, 41]. Artifact size curves (Schick’s debitage size distribution) [42, 43] were also analyzed, as another useful proxy to evaluate water disturbance.

Due to the lack of a standardized typology for Chinese Early Paleolithic stone tool assemblages [44–47], and since no Large Cutting Tools have been reported at FL, we followed primarily classification systems used in East African Oldowan assemblages [41, 48–54]. Each artifact was initially classified into the basic technological categories proposed by Isaac [52] and Isaac et al., [53], i.e., flaked pieces, detached pieces (or debitage), pounded pieces, and unmodified material (i.e., lithics which show no evidence of human modification). In order to provide additional descriptive details, flaked pieces were further classified into cores and retouched flakes following Leakey [49], Toth [50, 51], Kuman [55], and de la Torre and Mora [54]. Detached pieces were classified as debitage following criteria outlined by Leakey [49]. These include whole flakes, flake fragments, and angular fragments [50, 51, 54, 56, 57]. Flake types defined by Toth [50] were used to quantify cortex and examine flaking stages.

Typologically, we classified FL cores as either test cores, choppers, discoids, polyhedrons, or core scrapers. To evaluate reduction strategies, flaking methods followed schemes presented by de la Torre et al., [58], and expanded by de la Torre and Mora [54], and de la Torre [41]. This system is based on the location (unifacial, bifacial, multifacial), direction (e.g., unidirectional, bidirectional, centripetal), and angle (simple, abrupt) of flake removals. Retouched flakes are defined as those showing secondary removals that are normally less than 2 cm long, and suggesting edge shaping.

## Results

### The integrity of the FL site

The FL site was deposited in a lake margin environment where severe hydraulic disturbance is not apparent. For example, TOK sediments are composed primarily of very fine sand and coarse silt (53.70%) to fine silt to clay (37.07%), with a low percentage of relatively coarse particles. In this trench, archaeological layers are finer-grained than the other deposits, with 95.27% of very fine sand and coarse silt to clay, and a low percentage of coarse particles of fine sand to granules and small pebbles (4.73%), typical of lacustrine floodplains (see details in supporting information S1 Table). Therefore, no sedimentary evidence is currently available to suggest the occurrence of high-energy depositional events during the formation of the archaeological assemblage.

**Fig 4:**
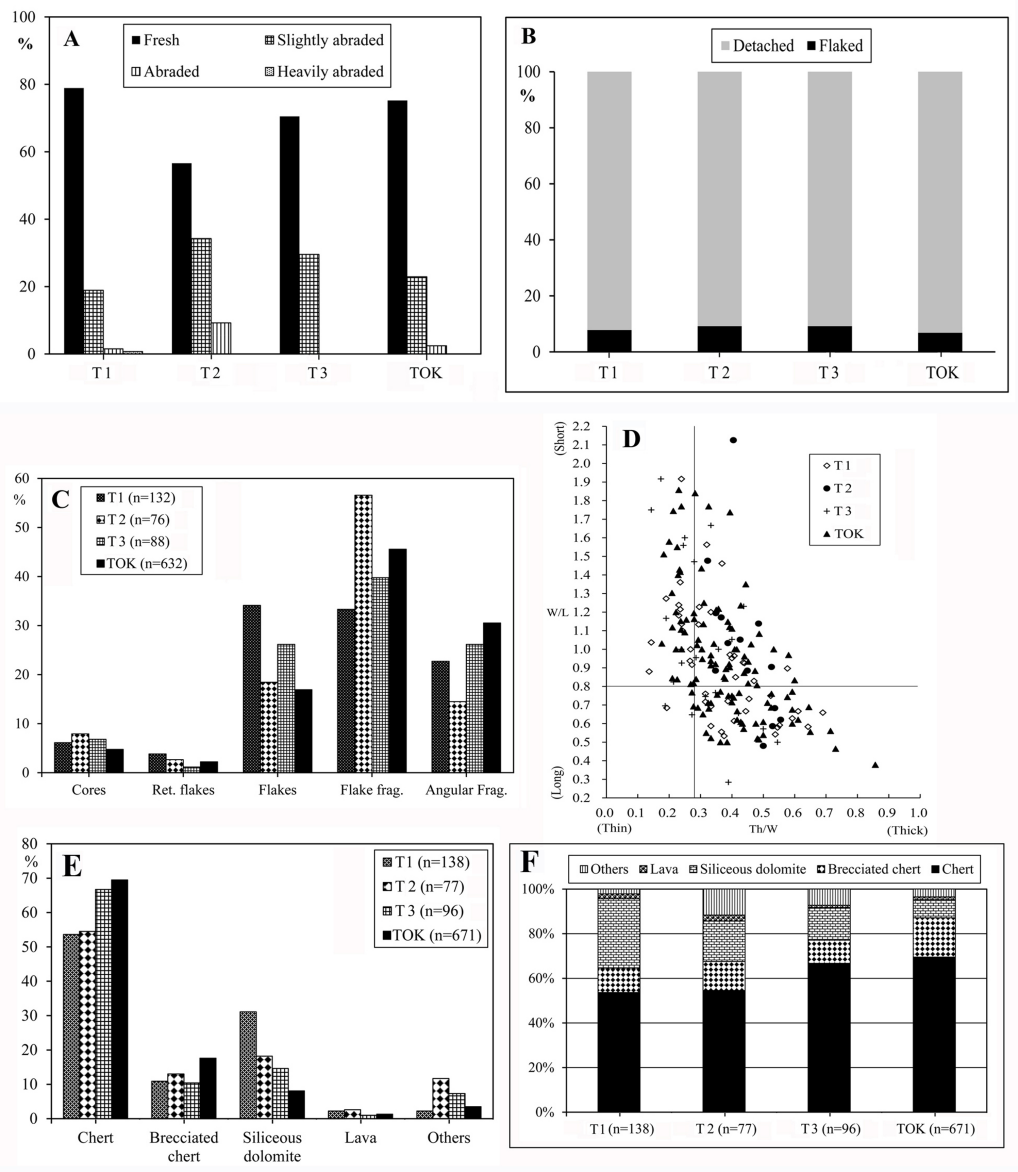
A) Stone tool abrasion in the FL assemblages. B) Ratios of flaked pieces: detached pieces. Unmodified blocks are excluded. C) Percentage of main lithic categories in the FL assemblages. Unmodified blocks are excluded. D) Shape of whole flakes in the FL assemblages, based on Width/Length, and Thickness/Width ratios. E) Distribution of raw materials in the FL site complex. F) Different raw materials exploited in the FL assemblages.

Artifact condition also suggests that the FL assemblages are mostly in primary context. As shown in Fig 4A, T1, T3, and TOK contain high percentages of fresh (78.9%, 70.5%, and 75.2%) and slightly abraded (18.9%, 29.5%, and 22.9% respectively) artifacts, whereas percentages of abraded and heavily abraded artifacts in T1 and TOK (2.2% and 2.4% respectively) are minimal, and no abraded or heavily abraded artifacts appear in T3. It should be noted that T2 shows a slightly different pattern, with 56.6% of fresh and 34.2% of slightly abraded artifacts, a lower percentage (9.2%) of abraded artifacts, and no heavily abraded artifacts. Therefore, the abrasion index suggests that most assemblages in T1, T3, TOK and (to a lesser extent) T2, were buried in primary context with minimal fluvial disturbance.

Intra-assemblage category ratios also support this view; as shown in Table 2, detached artifacts substantially outnumber flaked pieces. Fig 4B illustrates this point, highlighting the proportion (over 90%) of detached versus flaked pieces.

**Table 2:** Breakdown of lithic categories in the FL site complex. Surface and stratified artifacts are included.

	T1		T2		T3		TOK	
Category	N	%	N	%	N	%	N	%
Cores	8	5.8	6	7.8	6	6.3	30	4.5
Retouched flakes	5	3.6	2	2.6	1	1.0	14	2.1
Flakes	45	32.6	14	18.2	23	24.0	107	15.9
Flake fragments	44	31.9	43	55.8	35	36.4	288	42.9
Angular fragments	30	21.7	11	14.3	23	24.0	193	28.8
Unmodified materials	6	4.4	1	1.3	8	8.3	39	5.8
Total	138	100	77	100	96	100	671	100

**Fig 5:**
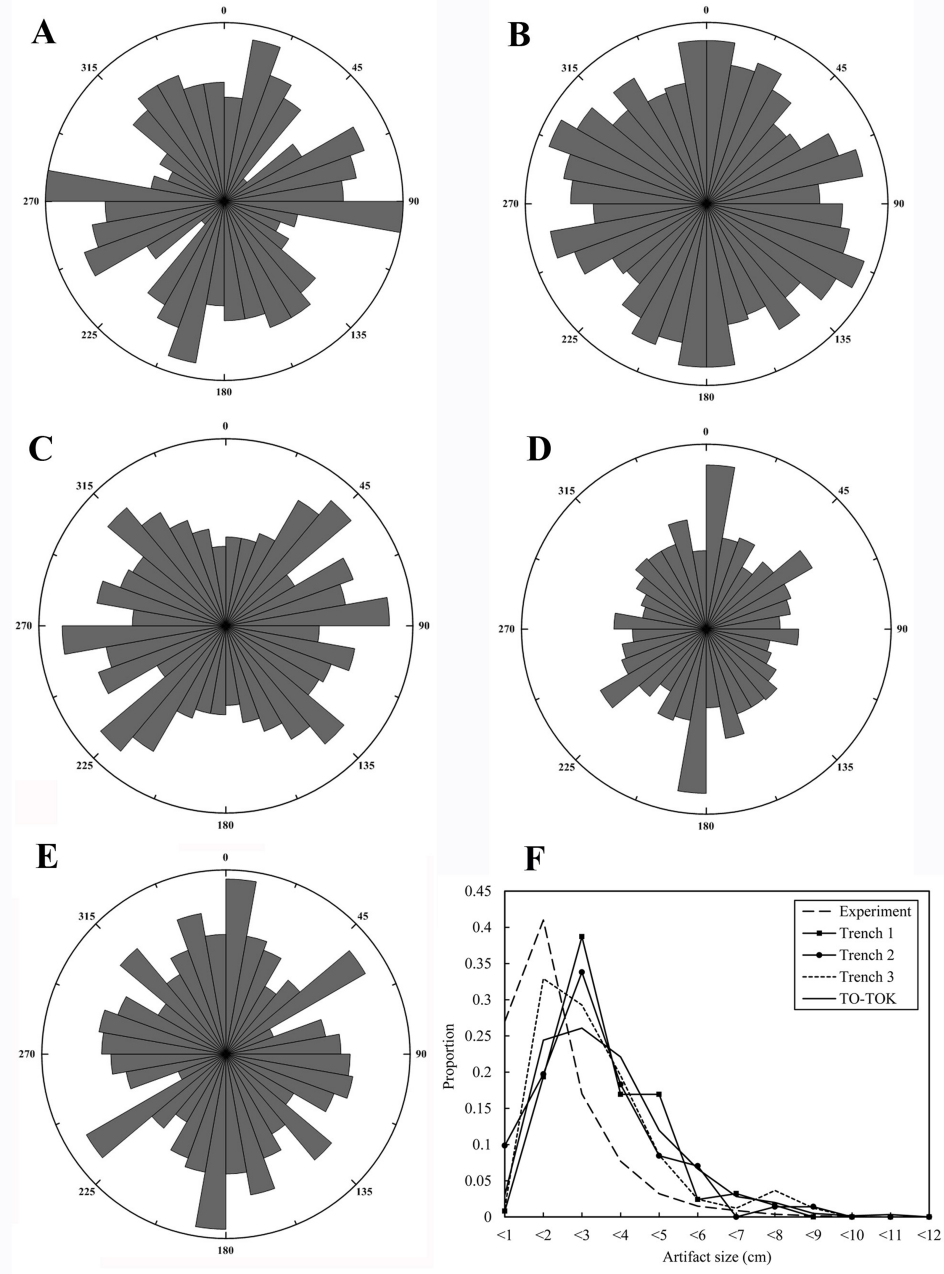
Rose diagrams of FL trenches. A) T1 stone tools and fossils (N = 138), B) T 2 stone tools and fossils (N = 547), C) T 3 stone tools and fossils (N = 450), D) TOK fossils (N = 716). E) TOK stone tools (N = 447). F) debitage size distribution patterns (T1, N = 124; T2, N = 71; T3, N = 82; TOK, N = 602), with reference to Schick’s (1986) experimental curve.

Artifact size curves also inform the degree of fluvial disturbance; debitage size patterns of FL trenches (Fig 5F) diverge from Schick’s experimental curves [43]. While her experimental debitage size distribution is dominated by material smaller than 20 mm (68%), frequencies at FL trenches are lower (20.0%, 29%, 34%, and 27%, for Trench 1, 2, 3, and TOK, respectively). FL artifact size distribution patterns show a peak in the 2–4 cm interval, which suggests that water flow may have washed out some of the lighter materials.

Fig 5A–5E compiles rose diagrams of lithics and fossils from the four trenches. Orientation patterns from T1, T2, and T3 are random, while TOK artifacts show a slightly preferred N-S trend. Nonetheless, in general orientation diagrams do not suggest heavy fluvial disturbance for the FL assemblages.

**Fig 6:**
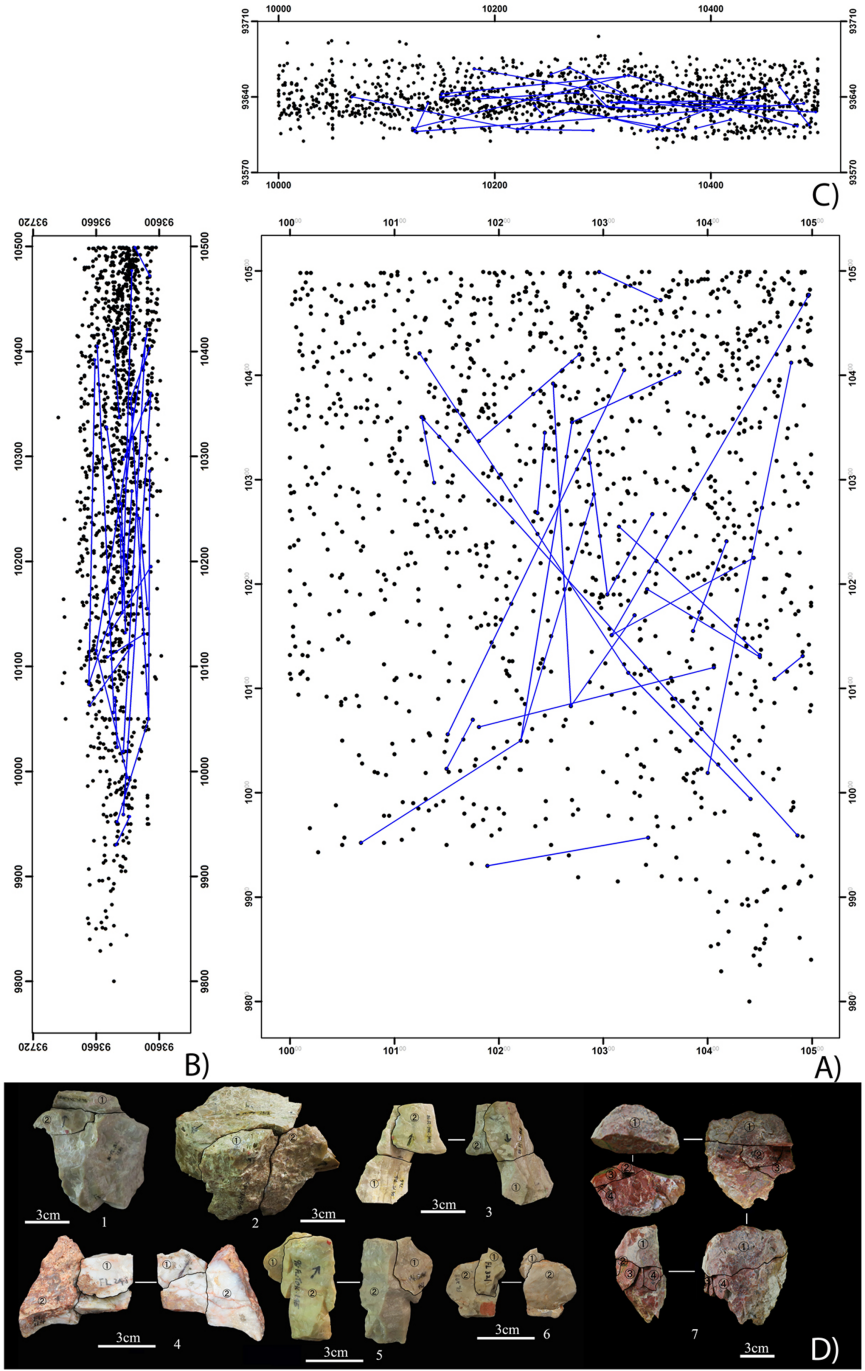
A-C) Plan view (A), and sagittal y, z (B) and transversal x, z (C) cross-sections of refit connections in TOK. D) Examples of refit sets in the FL assemblage.

Distribution patterns of refitted pieces help the evaluation of site formation processes at FL. Although several trenches yielded refits (see Fig 6D), given the higher density and abundance of artifacts at TOK, this trench is used here as a reference. In TOK, 44 pieces were refitted corresponding to 18 conjoining sets (Fig 6A–6C). Most refit sets (n = 12) are formed of 2 pieces, but several (n = 5) include 3 pieces, and one set contains 5 artifacts. The average horizontal distance between conjoining artifacts is 1.54 m, with the longest distance being 3.93 m, and the shortest 3 cm. As shown in Fig 6B and 6C, refit lines are generally flat in cross section, and have an average vertical distance of 18 cm. Overall, refit set dynamics indicate consistent spatial relationships of artifacts across the surface of the trench, and a discrete vertical dispersion of conjoining pieces.

In summary, lines of evidence discussed in this section such as artifact abrasion, size curves and orientation patterns, suggest that post-depositional disturbance was negligible. Such disturbance was probably limited to low energy sheet wash across the lakeshore setting that may have removed part of the small fraction of archaeological assemblages, but which did not alter significantly the original configuration of the FL site.

### The FL bone assemblage

**Fig 7:**
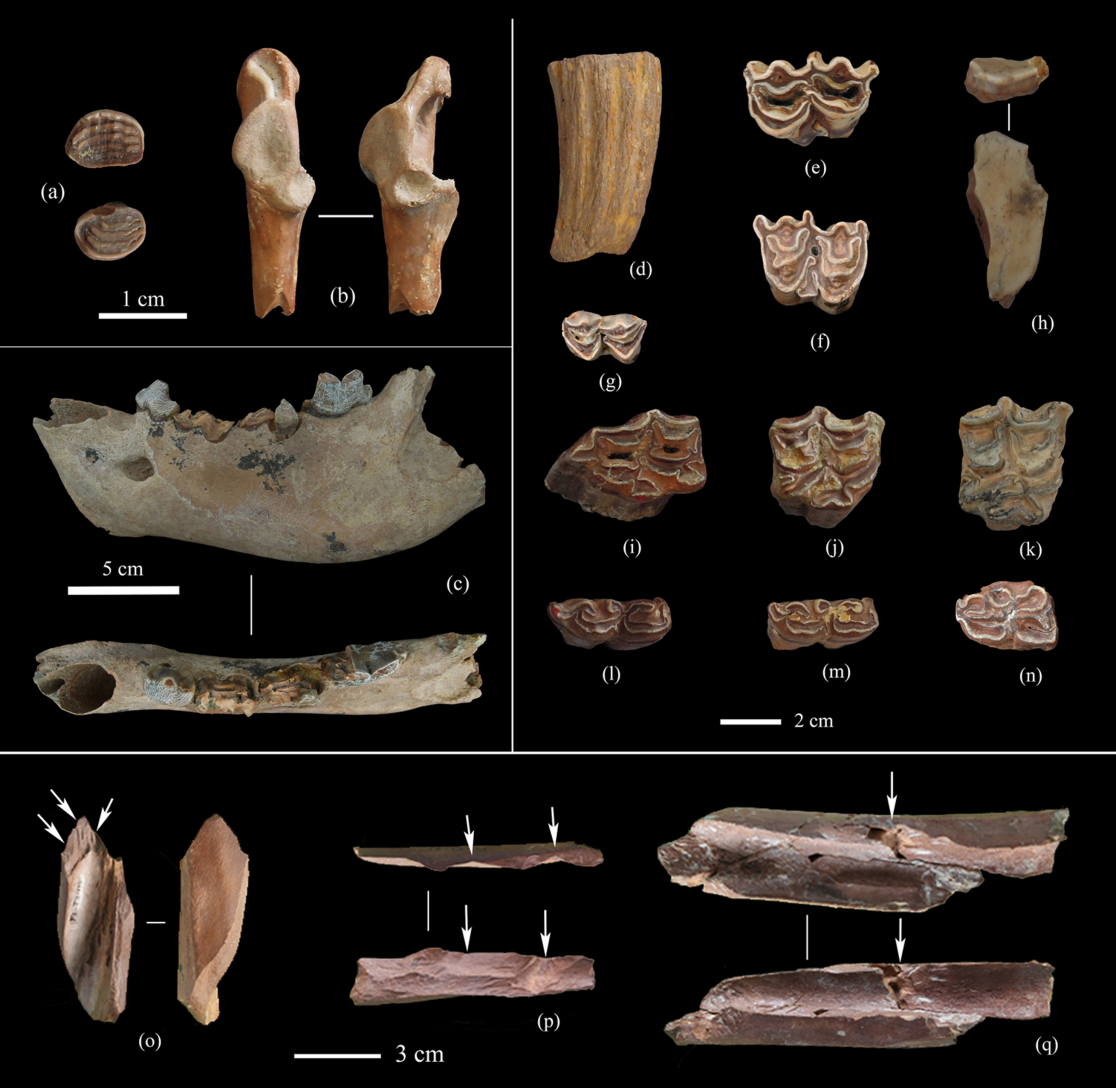
Examples of FL fossils. (a) Fish pharyngeal teeth (T2), (b) left proximal bird coracoid (T2), (c) *Pachycrocuta licenti*, left mandible fragment (T2); (d) *Bison palaeosinensis*, horn core fragment (TOK), (e) Bovidae gen. et sp. indet., lower m1/2, left (T3), (f) Bovidae gen. et sp. indet., upper M1/2, right (T3); (g) *Cervus sp*., lower m1/2, right (T3), (h) Rhinocerotide gen. et sp. indet., tooth fragment (T3), (i-k) *Equus sanmeniensis*, upper cheek teeth (i-T3, j-TOK, k-TOK), (l-m) *Equus sanmeniensis*, lower cheek teeth (l- TOK, m- TOK), (n) *Proboscidipparion sp*., lower molar, right (3), (o-q) Fresh fractures on bones (o-T3, p-T2, q- TOK).

Over 2,300 bones were recovered during excavation, of which more than 80% consist of post-cranial fragments (mostly limb bones), and 20% are dentition and isolated teeth. Fossils are very fragmentary and only 11 body parts were recognized, of which 10 species were determined (Table 3 and Fig 7). Macromammals are dominated by ungulates; Equidae are the most common, followed by Cervidae. Carnivores are scarce and micromammals have not been identified, although fish and bird remains are present.

**Table 3:** Summary of taxonomic groups, diet, body parts, and suggested environments represented in the FL bone assemblages.

Class	Taxon	Body parts	Environment
Fish	\	pharyngeal teeth, gill cover frag.	W
Birds	*Struthio* sp. \	egg shells	SS
		coracoid frag.	
Carnivores	*Meles leucurus?*	canines	F
	*Pachycrocuta licenti*	mandible frag., cheek teeth	OG,SS
	Canidae gen. et sp. indet.	canines	OG,SS
Perissodactyla	*Equus sanmeniensis*	cheek teeth, metacarpal frag.	OG,SS
	*Proboscidipparion* sp.	cheek teeth	OG,SS
	Rhinocerotide gen. et sp. indet.	cheek tooth frag.	OG,F
Artiodactyla	*Cervus* sp.	cheek teeth	OG,F
	*Bison palaeosinensis*	horn core frag., cheek teeth, radius and ulna frag.	OG,F
	Bovidae gen. and sp. indet.	cheek teeth, phalange, metacarpal frag.	OG,F

Environment: W = Water; OG = Open Grassland; SS = Sparse Steppes; F = Forest

In contrast to the “Classic Nihewan Fauna” [19], the FL faunal assemblage lacks species such as *Coelodonta Nihowanensis*, *Canis chihliensis* and *Spirocerus*, hindering comparison of FL with other sites in the Nihewan basin. Rodents are good chronological indicators in nearby sites such as Majuangou, Xiaochangliang, and Donggutuo [59, 60], but they are absent in FL. The presence of both *Equus* and *Proboscidipparion* at FL is consistent with other early Pleistocene sites such as Ruicheng, Wucheng, Xiaochangliang, and Shanshenmiaozui, where coexistence of those two Equidae is attested [60–62]. The FL species list is also similar to that of the Banshan site [63], dated at 1.32 Ma [11].

The Nihewan Fauna is often considered as the typical forest-steppe community of the early Pleistocene in North China [64]. In the specific case of the FL assemblage, ostrich and Equidae suggest the presence of large open temperate grasslands, while Cervidae indicate an interphase of forest and grassland, and fish remains point to the presence of nearby water. In summary, the FL settings would likely have included sparse shrubs, open plains and steppes, and certain areas with water.

As with other early Pleistocene archaeological sites in the Nihewan Basin, identification of bone breaking patterns and surface modification at FL requires taphonomic investigation to assess the extent of human intervention on the fossil assemblages. We have observed varied weathering stages of FL fossils, which would seem to imply several episodes of bone accumulation. Nevertheless, our preliminary taphonomic analysis of the FL fossil assemblage has identified green fractures and percussive notches indicative of human action on the bone assemblage (Fig 7).

### The FL lithic assemblages

#### Assemblage composition

The size of the Trench 1 lithic assemblage discussed in this paper (n = 138) is similar to Xie’s [39] original recount (n = 130). Cores, retouched flakes, flakes and flake fragments (see dimensions in Table 4) are well represented (Fig 4C); flakes (N = 45, 32.6%) and flake fragments (N = 44, 31.9%) predominate across technological categories (Table 2), with a flake/core ratio of 11.1.

**Table 4:** Size (mm) and weight (grams) of the main lithic categories in the FL assemblages.

		T1		T2		T3		TOK	
		Mean	S.D.	Mean	S.D.	Mean	S.D.	Mean	S.D.
Cores	Length	75.6	13.1	68.2	19.0	46.17	19.2	79.7	32.9
	Width	70.6	17.3	61.3	18.9	43.0	18.5	67.9	28.3
	Thickness	51.9	14.7	45.5	14.3	32.5	17.42	54.5	22.8
	Weight	381.9	197.7	266.0	186.3	145.2	254.1	489.9	645.7
Retouched flakes	Length	26.0	3.1	59	31.1	26		32.7	8.11
	Width	36.2	5.9	34.5	9.2	31		37.9	8.3
	Thickness	13.0	3.0	26.5	20.5	10		14.7	4.1
	Weight	12.4	2.6	151.5	194.5	7.1		18.9	12.1
Flakes	Length	32.1	11.4	35.2	17.7	27.8	17.2	35.3	13.1
	Width	27.8	9.4	34.4	16.6	24.6	13.9	31.5	12.1
	Thickness	9.9	4.0	14.8	7.2	7.65	4.86	11.6	4.9
	Weight	9.4	7.9	31.9	50.5	8.7	14.4	14.9	20.4

The lithic collection of Trench 2 (n = 77) is the smallest of the FL assemblages. Flakes (N = 14, 18.2%) and flake fragments (N = 43, 55.8%) predominate (Fig 4C and Table 2), although T2 yielded the lowest flake/core ratio (9.5) of the FL lithic assemblages. About the Trench 3 lithic assemblage (n = 96), flakes (N = 23, 23.9%) and flake fragments (N = 35, 36.5%) predominate (Fig 4C), and the flake/core ratio is 9.5.

The TOK lithic collection (n = 671) is the largest of the FL lithic assemblages. Flake fragments (N = 288, 65.6%) predominate, followed by angular fragments (n = 93, 28.8%) and flakes (N = 107, 24.4%) (Fig 4C). TOK has the highest flake/core ratio (13.2) of the FL lithic assemblages.

Further details on the lithic assemblage composition of each trench is available in supporting information S1 Appendix.

#### Raw materials

The FL assemblages include chert, various lavas and basement rocks, which were locally available to hominins in the vicinity of FL in the Cenjiawan Platform. Chert is the main raw material, probably derived from Precambrian rock outcrops about 200–500 m to the north and northeast of FL. Siliceous dolomite, the main rock type of this Precambrian rock system, appears in a chert-bearing bed in bands and as irregularly-shaped nodules. This rock system underwent secondary fracture transformation that resulted in the brecciated structure of the chert and siliceous dolomite. The chert is fine-grained silica-rich microcrystalline, cryptocrystalline or microfibrous. It varies greatly in color, but is often brown, gray, grayish brown, or rusty red. Siliceous dolomite is also fine-grained, gray and grayish white. Some chert exhibits internal flaws, fractures and a brecciated structure, which decrease its flaking quality. This type of chert is different from the relatively high-quality fine-grained chert, and is given the name of “brecciated chert” in this paper.

Lavas used for tools were probably derived from the Jurassic volcanic system located 100 m west and 500 m east of the FL site. The most frequently used lava is medium to dark grey, fine-grained, and either aphyric or slightly porphyritic; basalt, andesite and trachy-andesite are the most common types.

The basement rocks used by hominins include quartz, quartzite, and granite gneiss, which were extruded by Jurassic volcanic eruptions. The quartz is colorless or white and shows poor conchoidal properties. Nearly all the quartzite is white, pale yellow, and grey, very coarse-grained and displays similar fracture properties to quartz. A very few artifacts are of granite gneiss, which is coarse-grained and dark red and grey. Basement rock types are less common across the landscape than Jurassic lavas.

Overall, chert is generally the most suitable rock for flaking, followed by siliceous dolomite and lava. Brecciated chert and the basement rocks (i.e. quartz, quartzite and granite) are of relatively poorer quality. Chert and brecciated chert are the most abundant around the FL site, and are usually present as blocks or in bands, which weather into smaller pieces suitable for human collection. Siliceous dolomite, lava and other materials are relatively rare, were usually preserved as cobbles in the paleo-lake margin setting, and readily available to FL knappers.

Fig 4F shows distribution of raw material across FL trenches. Chert and brecciated chert are the most common raw materials in TOK (87.1%), in T3 they represent 77.1%, 67.5% in T2, and 64.5% in T1. Trench T1 has the highest percentage (31.1%) of siliceous dolomite cobbles among the FL lithic assemblages, while T2 shows the highest proportion (11.7%) of quartz, quartzite, and granite, followed by T3 (7.3%), and 4% in T1 and TOK. FL knappers rarely used lava rocks, which are below 3% in all trenches.

#### Knapping skill and reduction sequences

Core morpho-types are shown in Table 5. Knapping techniques are uniform across the four trenches and appear to be limited to freehand, hard-hammer, direct percussion, typical of Early Pleistocene Oldowan- Mode 1 sites. There is no evidence to suggest the presence of bipolar, anvil, or throwing techniques.

**Table 5:** Core morpho-types of the FL lithic assemblages.

	T1		T2		T3		TOK		Total	
Core type	N	%	N	%	N	%	N	%	N	%
Test core	2	25.0	1	16.7	1	16.7	1	3.3	5	10.0
Unifacial chopper	1	12.5	2	33.3	0	0	2	6.7	5	10.0
Bifacial chopper	1	12.5	0	0	1	16.7	2	6.7	4	6.0
Unifacial discoid	1	12.5	0	0	0	0	0	0	1	2.0
Bifacial discoid	0	0	0	0	0	0	4	13.3	4	8.0
Core scraper	2	25.0	2	33.3	3	50.0	11	36.7	18	36.0
Polyhedron	1	12.5	1	16.7	1	16.7	10	33.3	13	26.0

Unifacial flaking is evident on 75% of T1 cores, 33.3% of T2 and T3 cores, and 23.3% of TOK cores (Table 6). Bifacial flaking is evident on 36.7% of TOK cores, 16.7% of both T2 and T3 cores, and 12.5% of T1 cores. Fifty percent of cores in T2 and T3 are multifacial, with a similar proportion for TOK (40%) and a lower frequency in T1 (12.4%). In summary, the FL core assemblage shows a relative preponderance of multifacial flaking (38.0%), followed by unifacial (36.0%) and bifacial (28.0%).

**Table 6:** FL flaking modes.

	Trench 1		Trench 2		Trench 3		TOK		Total	
Flaking mode	N	%	N	%	N	%	N	%	N	%
Unifacial	6	75.0	2	33.3	2	33.3	7	23.3	18	36.0
Bifacial	1	12.5	1	16.7	1	16.7	11	36.7	14	28.0
Multifacial	1	12.5	3	50.0	3	50.0	12	40.0	19	38.0
Sub-total	8	100	6	100	6	100	30	100	50	100

**Fig 8:**
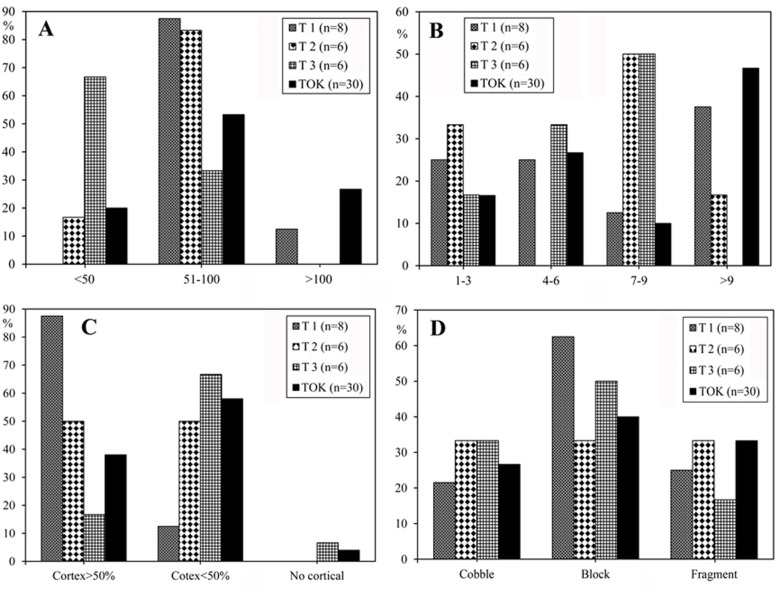
Core attributes in the FL assemblages. A) Core size ranges (mm). B) Number of flake scars on cores (scars per core average: Trench 1 = 8; Trench 2 = 7.2; Trench 3 = 6.3; TO-TOK = 8.8). C) Percentage of cortex. D) Core blank.

**Fig 9:**
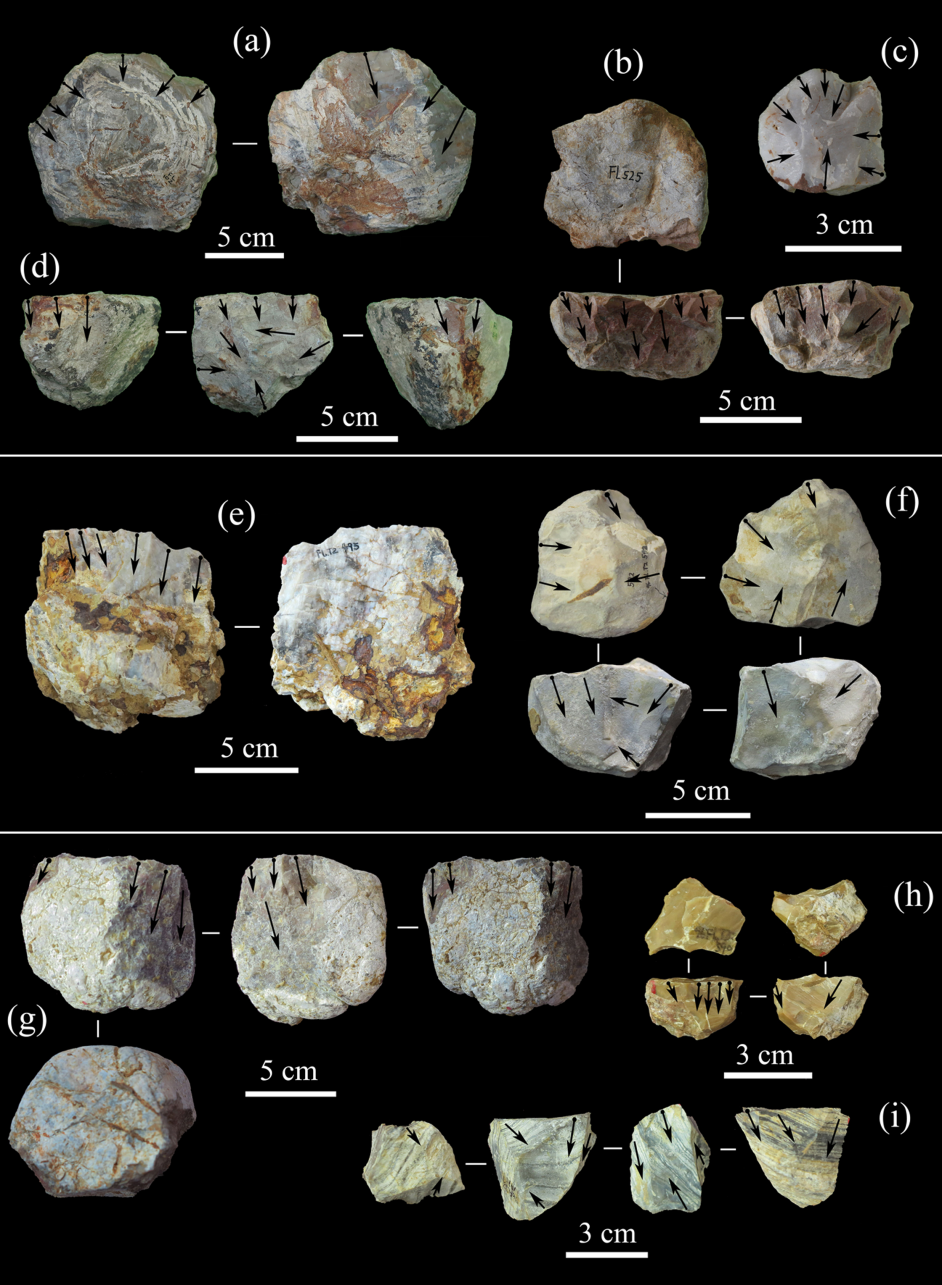
Selected cores from T1, T2, and T3. Upper (T1): (a) Bifacial chopper with BSP exploitation; (b) Core scraper showing UAP exploitation; (c) Unifacial discoid with UP exploitation; (d) Polyhedron with Multifacial exploitation. Middle (T2): (e) unifacial chopper with USP exploitation; (f) Polyhedron with Multifacial exploitation. Lower (T3): (g)-(h) Core scrapers showing UAP exploitation; (i) Polyhedron with Multifacial exploitation.

**Fig 10:**
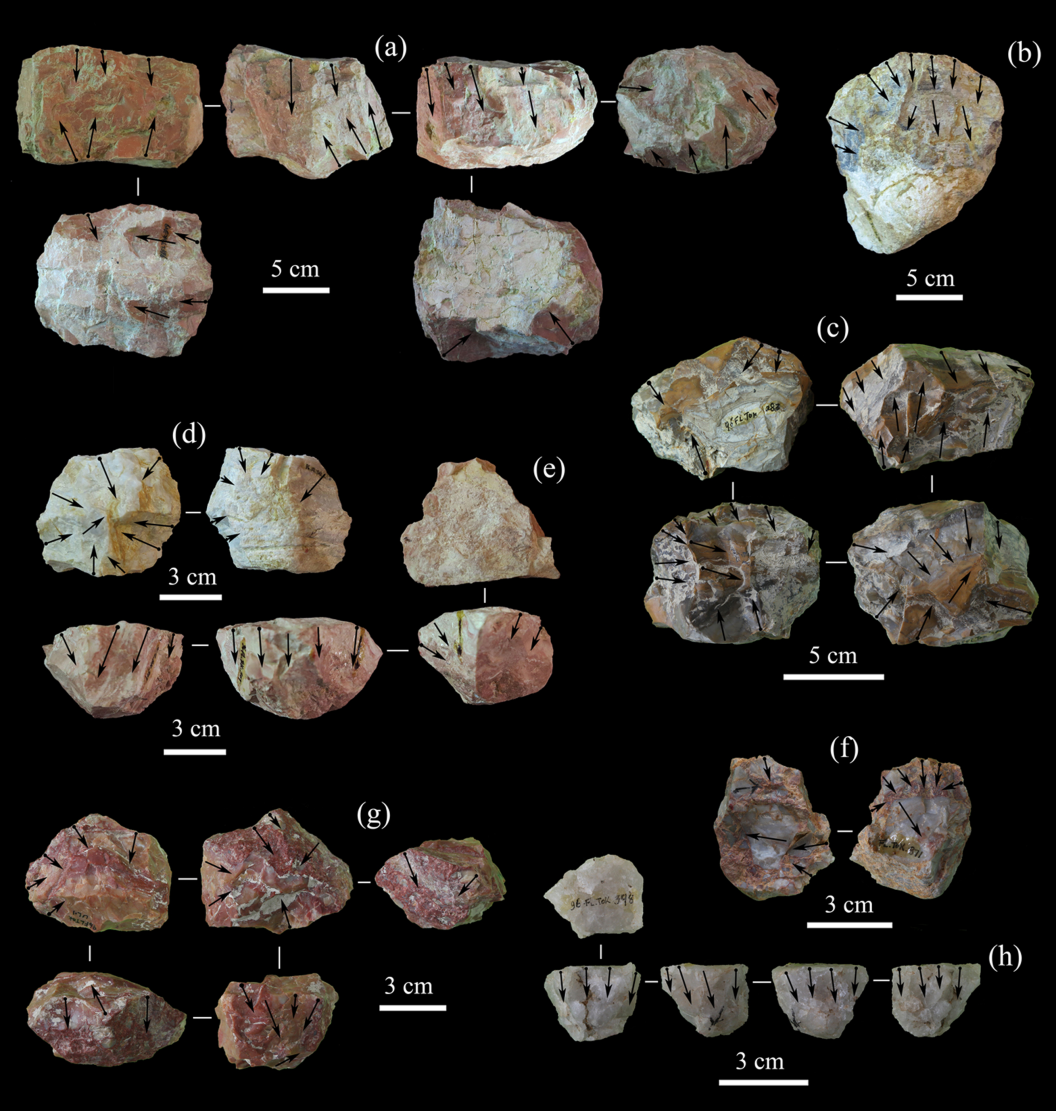
Selected cores from TOK. (a) and (c) Polyhedron with Multifacial exploitation; (b) Unifacial chopper with USP exploitation; (d) Bifacial discoid with BP exploitation; (e) Core scraper with UAP exploitation; (f) Bifacial discoid with BSP exploitation; (g) Bifacial discoid with BALT exploitation; (h) Core scraper with UAT exploitation.

Another trend of the FL assemblage is the rare occurrence of structured knapping methods. No polyhedral [65, 66] or flaking schemes that require hierarchization of flaking and striking surfaces (e.g. BHC) [41] are present. Table 7 shows that most FL cores (34.0%) result from multifacial knapping methods with no clear organization of flaking, and suggest an ad-hoc use of any available flaking angles. In general, flaking methods are expedient, show short reduction sequences, a lack of standardization in knapping methods, and great variability in size. Overall, FL knappers selected blocks and cobbles of various sizes (5–10 cm long), and followed relatively short sequences of flake removals (inter-assemblage average of 10.8 scars per core) before discard (Figs 8–10). Potentially, this behavior can be linked to the abundance and low quality of local raw materials, which might explain the expedient flaking patterns observed in all FL trenches.

**Table 7:** Absolute and relative frequencies of FL core knapping methods.

	T1		T2		T3		TOK		Total	
Flaking methods	N	%	N	%	N	%	N	%	N	%
TC	2	25.0	1	16.7	1	16.7	1	3.3	5	10.0
USP	0	0	2	33.3	0	0	2	6.7	4	8.0
BSP	1	12.5	0	0	1	16.7	1	3.3	3	6.0
UAP	1	12.5	1	16.7	3	50.0	7	23.3	12	24.0
BAP	1	12.5	1	16.7	0	0	1	3.3	3	6.0
UAT	1	12.5	0	0	0	0	1	3.3	2	4.0
UP	1	12.5	0	0	0	0	0	0	1	2.0
BP	0	0	0	0	0	0	5	16.8	5	10.0
BALT	0	0	0	0	0	0	2	6.7	2	4.0
Multifacial	1	12.5	1	16.7	1	16.7	10	33.3	13	26.0

#### Core attributes

The number of flake scars (maximum dimension ≥10mm) on cores gives a minimum estimate of the number of flakes that have been removed from a core [50, 56]. 56% of FL cores have more than 6 scars (Fig 8B), and among those, TOK has the highest average (8.8), followed by T1 (8.0), T2 (7.2) and T3 (6.3). The amount of surface cortex on cores can also be used as a gross estimate of reduction intensity. Fig 8C shows that 38.0% of FL cores preserve over 50% cortex, 58.0% preserve less than 50%, and only 4.0% have no cortex. This suggests moderate flaking of cores, in which reduction rarely was intense enough to remove all cortical surfaces. Measurement of edge angles provide the potential functional qualities of core edges, as well as serving as an indication as to whether further reduction was feasible [50, 56]. FL core edge angles vary between 71° and 98°, with a mean for T1, T2, T3, and TOK cores of 84.3°, 82.7°, 80.5°, and 81.3° respectively, suggesting that most cores were still amenable to further reduction.

#### Flakes

**Fig 11:**
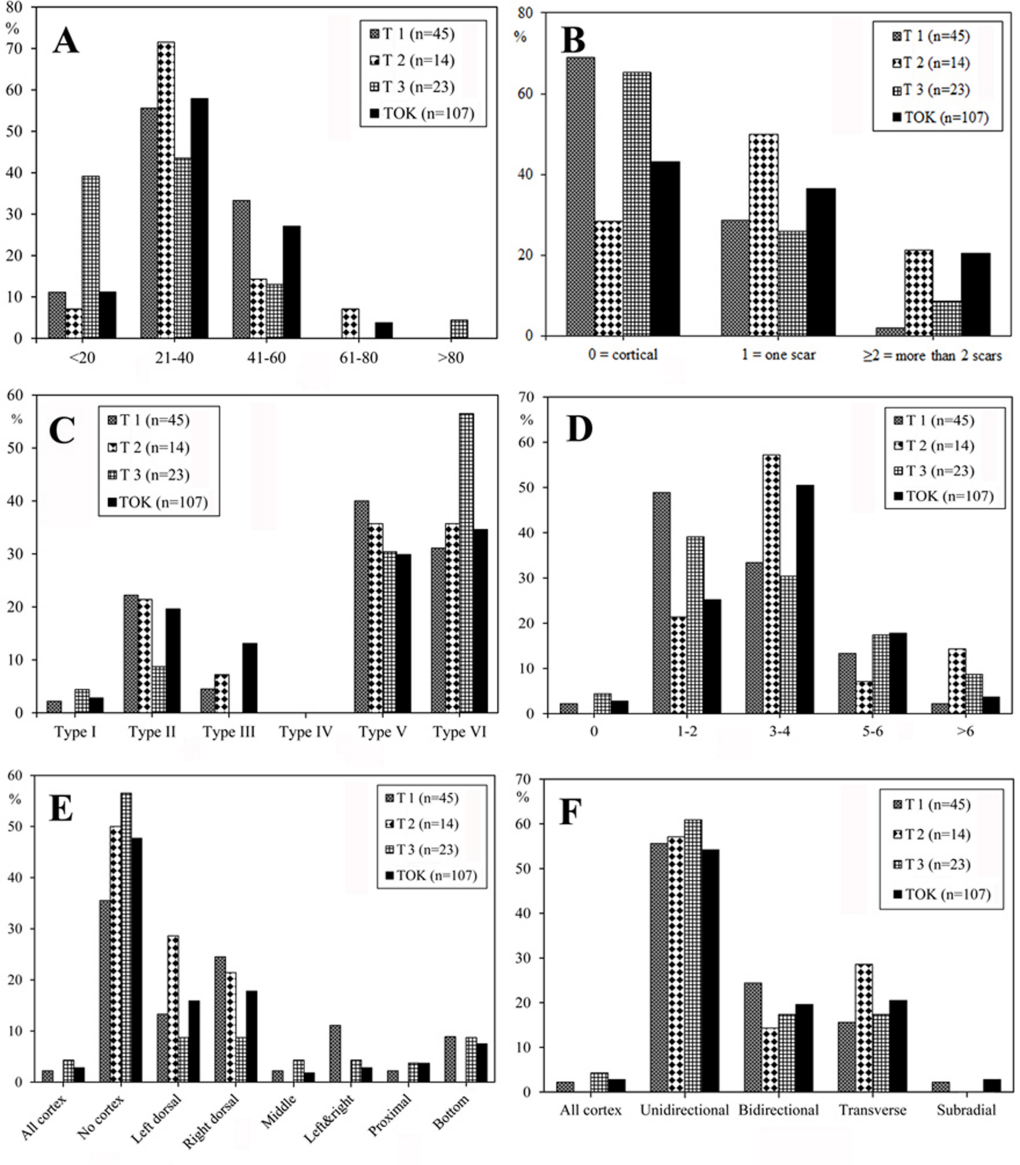
Flake attributes in the FL assemblages. A) Flake size ranges (mm). B) Number of scars on platforms. C) Percentage of cortex on dorsal faces and striking platforms, according to Toth’s (1982) types. D) Number of scars on dorsal face of flakes. E) Frequencies of cortical area on dorsal face of flakes. F) Frequencies of scar patterns on dorsal face of flakes.

**Fig 12:**
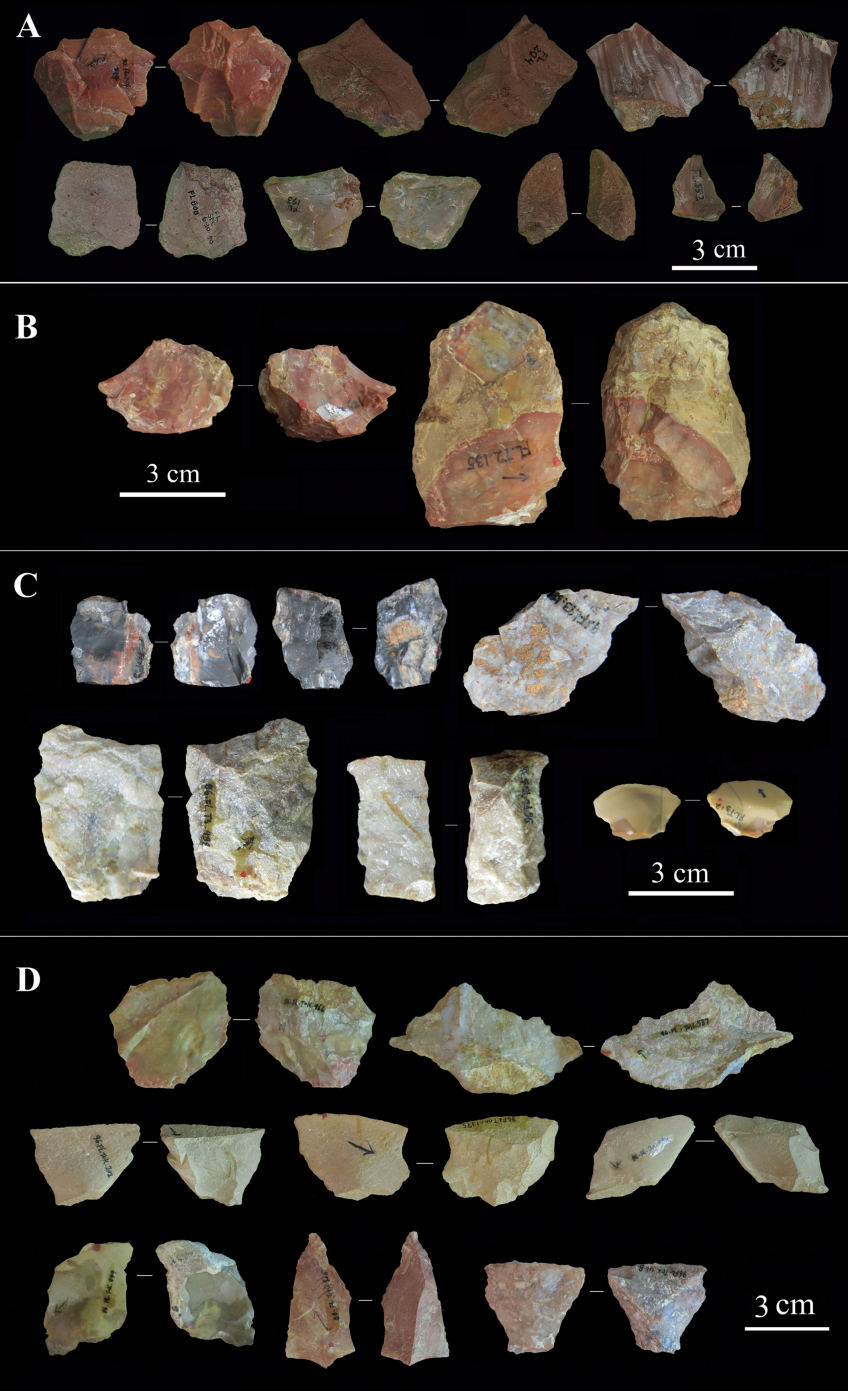
Selected flakes from T1, T2, T3, and TOK of the FL site. A) T1; B) T2; C) T3; D) TOK.

Toth’s flake types [50, 51] are good indicators of the prevalent mode of core reduction represented in an assemblage, and they provide the means for determining whether or not an assemblage is dominated by unifacial or bifacial flaking [51, 67, 68]. As shown in Fig 11C, FL flakes are characterized by high proportions of types VI (36.5%) and V (32.8%), moderate frequencies of type II (19.1%), and low proportions of types III (9.0%) and I (2.6%), with no type IV present. The higher percentage of types VI and V flakes indicates bifacial and multifacial flaking of cores, and suggests most flakes from FL result from knapping sequences after the roughing out stage. To some extent, these flake patterns are at odds with the short reduction sequences observed on cores (Fig 12). 56.6% of FL flakes range between 20mm and 40mm in length, 25.9% between 41mm and 60 mm, with no flakes larger than 100 mm. Width/length (W/L) and thickness/width (Th/W) ratios (see Fig 4D) indicate that, in general, flake shapes are moderately short and relatively thick.

Analysis of FL flake platform scars (≥1 mm) (see Fig 11B) shows that most (50.8%) are fully cortical, followed by unifacetted (34.4%), while the platforms with more than one scar constitute only 14.8% of the sample.

The number of dorsal scars on FL flakes shows relatively consistent patterns (see Fig 11D); 3–4 scars predominate (44.4%), followed by surfaces with 1–2 scars (32.3%) and 5–6 scars (15.9%), while those with more than 6 scars (4.7%) or no scars at all (2.7%) are rare. This pattern indicates that intense flaking sequences usually did not occur in the FL assemblages, thus supporting conclusions derived from core analysis.

The most common flake scar patterning is unidirectional (55.6%) (Fig 11E), followed by bidirectional (20.1%) and transverse (19.6%) patterns. Flakes with radial patterning only amount to 2.1% (Fig 11F), as do fully cortical flakes. Location of cortex on flake dorsal surfaces (Fig 11E) shows that flakes with non-cortical dorsal surfaces dominate (46.0%).

#### Retouched tools

**Fig 13:**
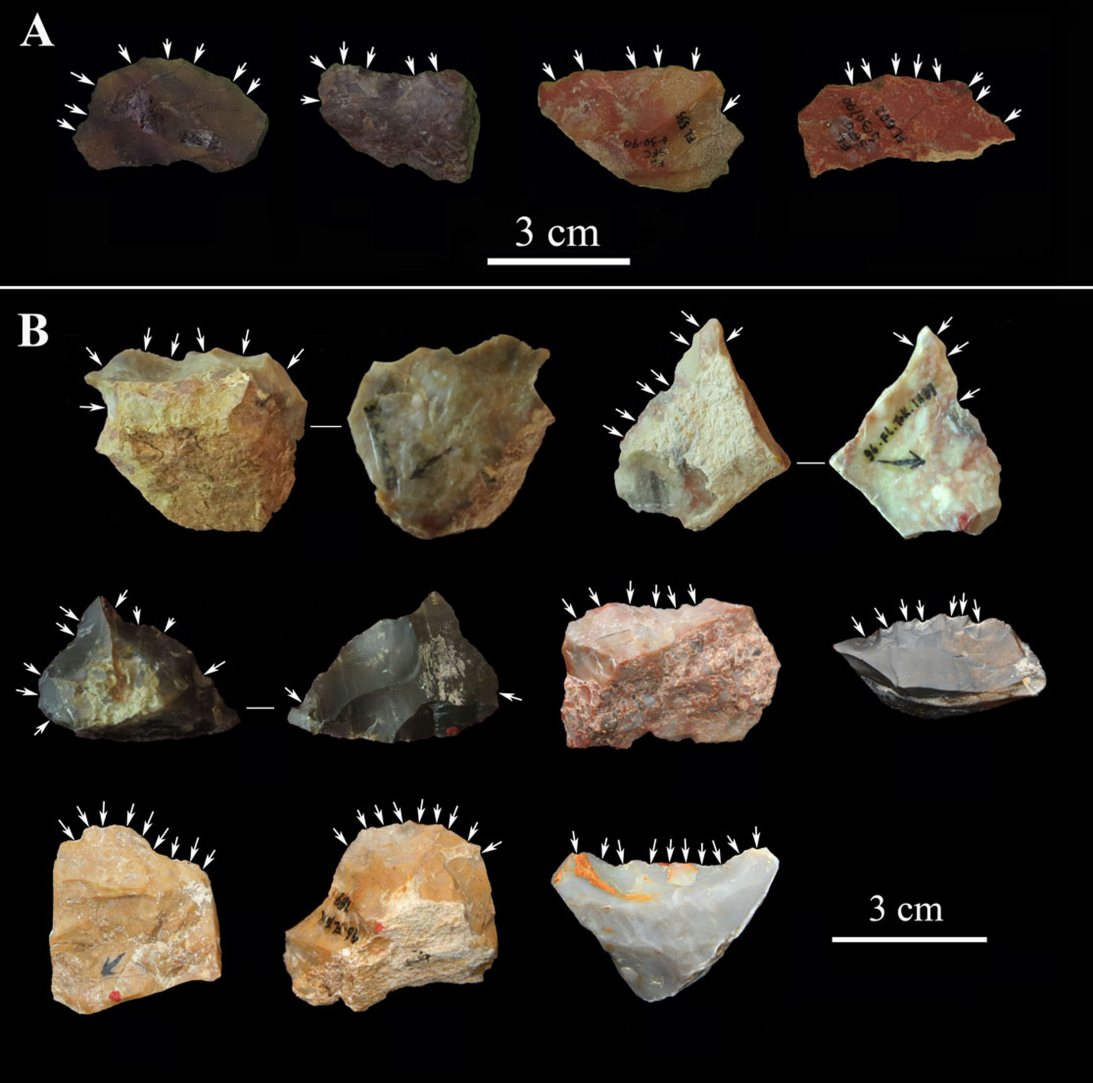
Selected retouched flakes from T1 and TOK. A) T1; B) TOK.

Albeit scarce, retouched flakes are present in all trenches, with an average proportion of 2.3%. Retouch is normally on flakes or flake fragments, and retouched tools average 33.4 mm in size. Retouch is casual in all FL assemblages, with no imposition of standardized shapes on blanks (see Fig 13).

## Discussion

### The FL site in the context of the Early Pleistocene archaeology of the Nihewan Basin

The nature of inter-site assemblage variability can provide relevant information in the reconstruction of hominin technological behavior [41, 51, 53, 56, 68, 69], which is an important goal of current research in the Nihewan Basin [26, 28]. Several tens of Early Pleistocene sites have been discovered in the Cenjiawan Platform [70, 71], and to date 12 sites have been reported (Table 8). The available magnetostratigraphic data documents human occupation of the Nihewan Basin between the termination of the Olduvai subchron and the Matuyama–Brunhes geomagnetic reversal, that is, between 1.77 and 0.78 Ma (Table 8). This considerable time span and the relatively large archaeological sample preserved, elicits a discussion on archaeologically relevant questions such as site resolution and early Pleistocene technological patterns.

**Table 8:** Summary of Early Pleistocene archaeological site contexts excavated in the Nihewan basin.

Site	Year excavated	Age (Ma)	Excavated area (m^2^)	Level thickness (cm)	Level stratigraphy	Number of items		Density of artifacts/m^2^	Modified bones without the?	References
						Lithics	Fossils			
MJGIII	2001–2002	1.66	85	50	Silty clay	443	1014	8.8	Y	[11]
MJGIII-G	2001	1.66	12	50	Clay silt and sand	50	871	4.17	Y	[25, 71]
MJDII	2002	1.64	40	36	Sandy silt	226	174	5.65	?	[11, 70]
MJGI	1993,2002	1.55	50	75	Clay silt	215	237	4.3	Y	[11, 70]
XCL1	1978	1.36	?	50–80	Silty sands	804	?	-	N	[9, 72]
XCL2	1990–1997	1.36	?	50–80	Silty sands	1258	?	-	N	[9,73–75]
XCL3	1998	1.36	16	80	Fine sand	901	3291	56.31	N	[73, 75]
DCL	2000	1.36	7	58	Silty sand	33	22	4.71	N	[76, 77]
BS	1990	1.32	2	40	Sand and gravels	95	130	47.5	N	[11, 63]
FL-T1	1990	1.2	17	50	Fine silt and clay	133	431	6.53	N	[70]; this paper
FL-T2	1996	1.2	18	160	Fine silt and clay	77	567	4.28	N	This paper
FL-T3	1996	1.2	12	195	Fine silt and clay	96	518	7.67	N	This paper
FL-TOK	1996	1.2	35	210	Fine silt and clay	671	871	19.11	N	[33]; This paper
CJW	1986,1992	1.1	40	10–35	Clay	1383	257	34.57	?	[27, 70, 78]
DGT1	1981	1.1	45	320	Clayey silt with gravels	1443	>1000	32.07	Y	[34, 79, 80]
DGT2	1997	1.1	12	40	Clayey silt	702	169	58.5	?	[34, 80]
HJD	1997	1.0	6	75	Silt with gravels	60	?	10	N	[81, 82]
ML	1985	0.8	20	40–60	Sand	121	?	6.05	N	[34, 71]

#### Site contexts of the Nihewan Basin archaeological sites

Identification of agents that contributed to assemblage formation is a constant concern in Early Stone Age research [36, 38, 42, 83–86] and the Nihewan sequence is no exception [87]. Present evidence indicates that all the Nihewan sites were buried along a paleo-lake margin [9–11, 26, 29–31], although details on the specific paleoecological setting of each site need to be refined.

Sedimentary contexts are varied among the twelve Nihewan sites listed in Table 8. FL and CJW contain the finest deposits (from clay to silty clay); MJG and DGT sediments are mainly sandy silt to silt clay, while other sites such as BS, ML, and HJD contain sand and sand with gravels indicating a relatively high energy context. Pending the publication of detailed geoarchaeological studies, sedimentary contexts suggest that DGT, BS, HJD, and ML sites were formed in fluviatile settings, while water disturbance at FL, CJW, MJG, and XCL sites was less significant.

Artifact density and the thickness of archaeological levels can also be used to evaluate site formation dynamics [49, 88, 89], and to discuss whether assemblages are the result of short duration single episodes of human occupation, or palimpsests with multiple, sequential depositional episodes, caused by human and non-human agents [90]. As shown in Table 9, thickness of the archaeological levels in the Nihewan sites varies greatly; archaeological units in MJGII and CJW are the best constrained vertically, while levels at FL-T2, FL-T3, FL-TOK and DGT are all more than 1 m in thickness. Other sites such as FL-T1, MJGI, MJGIII, MJGIII-G, XCL, DCL, DGT-T2 and HJD contain archaeological levels with 40–80 cm of thickness.

**Table 9:** Breakdown of lithic artifacts of Early Pleistocene sites in the Nihewan basin. UM: unmodified material.

	Lithic assemblages earlier than 1.3Ma															
	MJGIII-G		MJGII		MJGI		XCL1		XCL2		XCL3		DCL		BS	
	N	%	N	%	N	%	N	%	N	%	N	%	N	%	N	%
Cores	2	4.0	32	14.2	11	5.1	25	3.1	44	3.5	44	4.9	4	12.1	8	8.4
Ret. pieces	2	4.0	25	11.1	1	0.5	12	1.5	12	1.0	7	0.8	1	3.0	20	21.1
Flakes	13	26.0	85	37.6	51	23.7	47	5.8	232	18.4	143	15.9	11	33.3	28	29.5
Flake frag.	11	22.0	34	15.0	75	34.9	720	89.6	211	16.8	197	21.9	5	15.2	14	14.7
Angular frag.	22	44.0	50	22.1	77	35.8			558	44.3	414	45.9	12	36.4	25	26.3
Bipolar	-	-	-	-	-	-	-	-	170	13.5	56	6.2	-	-	-	-
UM	-	-	-	-	-	-	-	-	31	2.5	40	4.4	-	-	-	-
Total	50	100	226	100	215	100	804	100	1258	100	901	100	33	100	95	100
	Lithic assemblages younger than 1.3 Ma															
	FL-T1		FL-T2		FL-T3		FL-TOK		CJW		DGT1		HJD		ML	
	N	%	N	%	N	%	N	%	N	%	N	%	N	%	N	%
Cores	8	5.8	6	7.8	6	6.2	30	4.5	36	2.6	147	8.8	4	6.7	15	12.4
Ret. pieces	5	3.6	2	2.6	1	1.0	14	2.1	33	2.4	230	13.7	24	40.0	29	24.0
Flakes	45	32.6	14	18.2	23	24.0	107	15.9	178	12.9	505	30.1	12	20.0	24	19.8
Flake frag.	44	31.9	43	55.8	35	36.5	288	42.9	1134	82.0	419	25.0	-	-	10	8.3
Angular frag.	30	21.7	11	14.3	23	24.0	193	28.8			375	22.4	20	33.3	43	35.5
Bipolar	-	-	-		-	-	-	-	2	0.1	-	-	-	-	-	-
UM	6	4.4	1	1.3	8	8.3	39	5.8	-	-	-	-	-	-	-	-
Total	138	100	77	100	96	100	671	100	1383	100	1676	100	60	100	121	100

**Fig 14:**
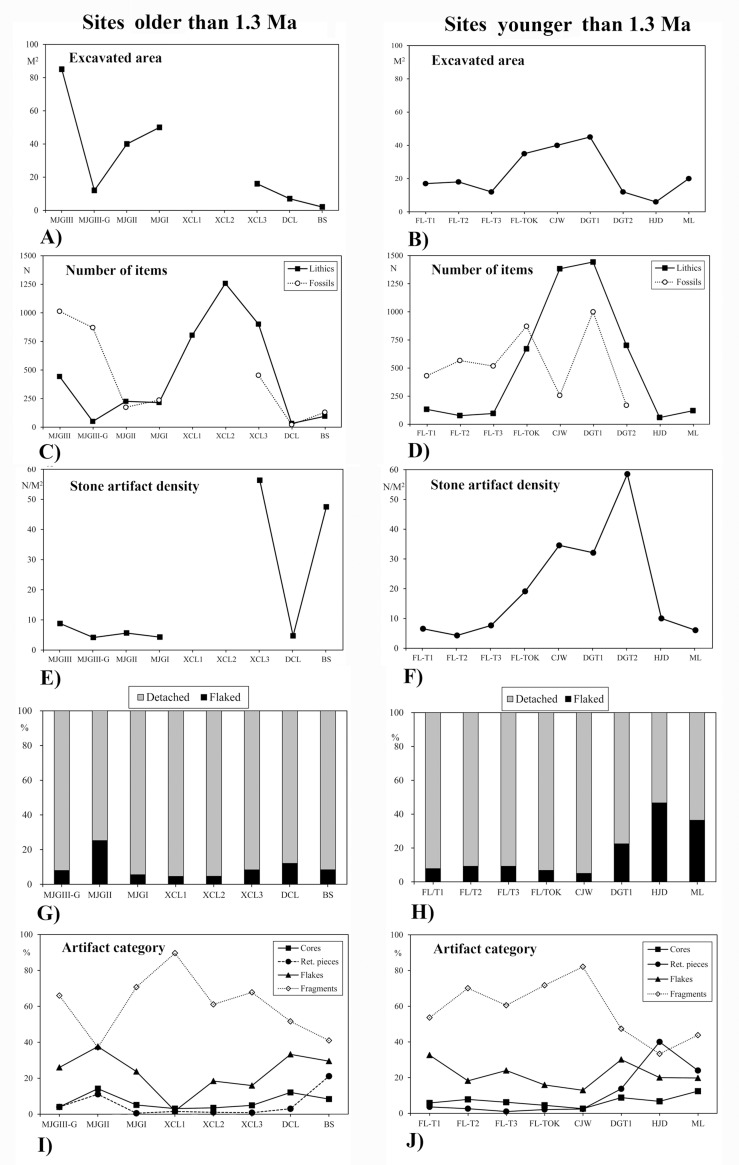
Comparisons of Early Pleistocene sites in the Nihewan basin. A) and B) Excavated area; C) and D) Number of items; E) and F) Stone artifact density; G) and H) Ratios of flaked pieces versus detached pieces; I) and J) Artifact category. All data from Tables 8 and 9.

Stone tool density (Fig 14E & 14F) also varies greatly across assemblages. Nine sites have low-density assemblages (less than 10 artifacts per square meter), in contrast to XCL3 and DGT2 (more than 50 artifacts per square meter). FL-TOK seems to present a middle ground, with close to 20 artifacts per square meter.

Except for the FL collections (this paper), no data is available regarding conditions of abrasion, frequencies of small flaking debris and fabrics of the Nihewan lithic assemblages, Although assessments of the spatial configuration of remains [91] and refits [27, 92] have been presented for CJW.

Notwithstanding, available data enables calculation of detached: flaked piece ratios, a proxy that has been used elsewhere to discuss assemblage integrity [36, 41, 42, 52]. Table 9 and Fig 14G & 14H show that MJGII, DGT and, particularly HJD and ML, contain relatively low proportions of detached pieces. Such a shortage of detached pieces can be linked potentially to significant post-depositional disturbance. In contrast, FL, MJGIII, MJGI, XCL, BS, and CJW assemblages all contain high proportions of detached pieces, whereas cores form consistently less than 10%, which can be used as a proxy to argue for a lesser post-depositional disturbance of the assemblages.

In summary, our comparison of the available data suggests that, to some extent, post-depositional processes affected all Nihewan assemblages, and should be considered when attempting to reconstruct hominin activities at the sites. In our opinion, archaeological occurrences from FL, CJW, MJGI, MJGIII, and MJGIII-G sites were less disturbed by post-depositional processes, whereas other sites such as HJD, ML experienced more significant hydraulic disturbance.

#### The Nihewan bone assemblages

Faunal remains constitute a large part of the Nihewan Basin assemblages, often outnumbering stone tools (Table 8, Fig 14C & 14D), although so far emphasis has been placed on taxonomic and paleoenvironmental aspects of collections, rather than on the vertebrate taphonomy and zooarchaeology of the Early Pleistocene sites.

As with the FL faunal assemblage described in this paper, fragmentary mammalian fossil bones and teeth (rodents, carnivores, elephantids, *Equus* sp., *rhinoceros* and *cervids*) were identified at MJGIII-G, as were eggshells of *Struthio* sp. [25, 93]. The vertebrate fauna in MJGIII includes *Elephas* sp., as well as molluscs [11, 25]. Mammalian fossils from archaeological units at XCL and DGT include *Palaeoloxodon* sp., *E*. *sanmeniensis*, *Coelodonta* sp., and *Gazella* sp. [72, 94]. Carnivores were also recorded: *Hyaena* sp. [72] at XCL, and *Canis* sp. at DGT [94]. No data was compiled by You et al., [72] on the number of fossils and MNI frequencies at DGT and XCL, which limited interpretation of the assemblages [29, 95, 96]. Recent research on the XCL faunal assemblages [97] proposes that hominin involvement in the formation of the faunal assemblage cannot be substantiated, given the lack of cut and percussion marks. This contrasts with MJGIII, where percussion marks are present on horse and cervid shaft bones, and interpreted as the result of hominin marrow extraction activities [11]. The DGT faunal assemblage may also contain cut marks (Schick and Toth, pers. comm. to Pei, 2000), but the assemblage awaits systematic taphonomic review.

#### Technological patterns in the Nihewan Basin sites

Recent studies of some of the Early Pleistocene lithic assemblages (e.g., XCL, DGT, MJGIII-G, CJW) (see Table 9) provide comparative data with which to discuss FL technology in the contexts of Nihewan basin technological strategies. As a whole, Early Pleistocene Nihewan lithic technology has often been characterized as a simple one, where cores are expediently knapped and small flake tools are made on locally available raw materials [26, 29, 75].

Pei and Hou [98] provided a general assessment of the formation mechanisms and geographic distribution of the different raw materials in the Nihewan Basin, but little is known about inter-site raw material variability in the Cenjiawan Platform. As discussed in this paper, FL hominins procured abundant, locally available, relatively poor quality brecciated chert, and siliceous dolomite of variable quality, with a minor input of other materials including fine -grained, high-quality chert, volcanic lava, and quartz. At MJGIII-G [25], XCL [28, 74–75, 96], and DGT [79, 80, 98], chert nodules and siliceous dolomites with embedded chert were also primarily selected for tool manufacture. As such, XCL hominins selected chert almost exclusively (98.3%), with very low proportions of vein quartz, basalt, and quartzite [28, 74, 75]. Gao et al. [25] referred to the chert in MJGIII-G as chert-like quartzite, which is also the main raw material at this site. The same pattern is observed in DGT, in which chert dominates (96.6%), with low percentages of basalt, quartz, and indurated sandstone [96, 99]. In the large CJW collection, 92.7% of stone tools are of fine-grained chert or brecciated chert [27, 70]. Although no frequencies have yet been published for the DCL, BS and HJD assemblages, chert or brecciated chert are also known to dominate.

In summary, there is a clear pattern where chert is consistently chosen in all Early Pleistocene Nihewan sites. In this regard, it should also be noted that researchers often give different names to the same rock type, and our own investigations in the Cenjiawan Platform [98] highlight that chert and brecciated chert can be found either as nodules or embedded within siliceous dolomites and similar rocks. Although Shen and Wei [100] indicate that ML and CJW hominins might have intentionally selected good-quality raw materials, no specific details were provided. According to available evidence, it can be concluded that during the Early Pleistocene, Nihewan hominins did not generally select higher quality raw materials. Instead, they selected locally abundant chert nodules of poor knapping quality; as discussed elsewhere [28, 29, 75, 96], this chert is often riddled with impurities that cause nodules to fracture in unpredictable ways. This likely explains why most assemblages show an extremely high incidence of angular fragments, short reduction sequences, and low standardization of flaking schemes.

With regards to flaking techniques across the Nihewan sites, refitting studies at FL and CJW [27, 92] provide some of the best support for the dominance of freehand percussion in the Early Pleistocene assemblages. Bipolar flaking is well attested in XCL2 and XCL3 [28, 75], but is poorly represented in other sites such as CJW [27, 28]. Although one cannot rule out that part of the relatively high number of detached pieces at DGT1-2 may be associated with bipolar flaking, this technique was not identified in Hou’s comprehensive study [99]. Work currently in progress on the DGT assemblages excavated in 2000 to 2001 has observed bipolar traits in some cores and flakes. No bipolar traits have yet been identified in the FL assemblage, which concurs with the absence of this technique in other sites noted in Table 9 such as MJG [11, 25, 70, 71], DCL [76], BS [63], HJD [81], and ML [71, 100]. Nevertheless, it should be noted that both freehand and bipolar methods can be applied on the same nodule, i.e., nodules could have been knapped initially with direct, freehand percussion, and resulting chunks further reduced through bipolar reduction [92]. Additionally, further studies of several collections studied by the same researcher/s may help reduce inter-analyst variance in the recognition of bipolar products (see discussion in Byrne et al. [101]).

Tables 8 and 9 and Fig 14A–14F show that the frequency of artifacts across sites differ greatly, which can be explained by variable density of remains per site (discussed above), and disparate size of excavation trenches. Nevertheless, Fig 14I and 14J show that percentages of intra-assemblage categories also vary when different sites are compared. Freehand flake fragments dominate all collections with the exception of MJGII and HJD. Apart from MJGII, DCL, and ML, freehand cores usually form less than 10% of the assemblages, and in MJGIII, XCL1, 2, 3, FL-TOK, and CJW their frequency is under 5%. Whole flakes exceed 30% at FL-T1, MJGII, DCL, DGT1 sites, but range between 10% and 20% at XCL2,3, FL-T2, FL-TOK, CJW, and in XCL1 only form 5.8% of the assemblage. Except for DGT1, HJD and ML, retouched pieces constitute the lowest frequency of artifact types. It should also be noted that pounded pieces such as hammerstones or anvils, are currently poorly known across the Early Pleistocene Nihewan assemblages.

Core forms and flaking methods are among the most important technological elements characterizing Early Stone Age assemblages. Recent studies of the Nihewan material [28] have begun to apply flaking schemes developed for African assemblages [41, 58], an approach which is also followed in the present paper. Judging from published results, choppers, polyhedrons, and core scrapers are the dominant core morpho-types. Despite limitations in the sample available for comparison due to the lack of a unified terminology, we deduce from the published reports that, as with the FL cores described in this paper, most Nihewan assemblages show a prevalence of simple and short flaking schemes (e.g., USP, BSP, UAP, and BAP). Nevertheless, refitting and attribute analysis has also recognized multiplatform knapping methods at XCL and CJW [75]. This type of core reduction involves continuously rotating the core to create new platforms suitable for flake removals, and cores continue to be exploited until near exhaustion [92]. In addition, Hou [99, 102] reported the presence of a more advanced type of wedge-shaped core forms at DGT, which produced predetermined small and elongated flakes, although Keates [26] remained unconvinced of the purported preparatory flaking stages in the DGT cores. Whatever the case, it is safe to state that, at present, structured core reduction techniques are an exception in the Nihewan assemblages, which are mainly simple and short.

One general pattern of the Nihewan reduction sequence is that all assemblages contain abundant small-sized flakes, especially FL-T1, MJGIII-G, MJGI, MJGII, DCL, DGT1, BS, FL-T3, and HJG. This is probably related to the poor quality of most raw materials, which readily shatter into irregular pieces [25, 26, 29, 92] and do not allow removal of large blanks. Despite the unreliability of raw materials and the short reduction sequences observed on cores, flakes in most assemblages are often non-cortical, and some preserve relatively high numbers of earlier scars on their dorsal surfaces. These features have led some researchers to suggest that flakes were extensively reduced during later stages of core reduction [27, 28, 100].

Retouched pieces in BS, HJD and ML sites exceed 20% percent of the assemblages, while in MJGII, DGT1 they range between 10% - 20%, and less than 5% in MJGIII-G, MJGI, XCL, DCL, FL, and CJW. No standardization is evident in flake retouching among sites older than 1.3 Ma. However, there seems to exist a different trend in assemblages younger than 1.3 Ma in sites such as DGT, CJW, and ML, where standard morpho-types, such as scrapers, notches, points, and denticulates, have been reported [27, 70, 79, 80, 100].

In summary, an overview of all the Early Pleistocene assemblages in the Nihewan Basin confirms the prevalence of a core and flake, Oldowan-like/ Mode 1 technology. Such technology was based on the procurement of relatively low quality chert and brecciated chert, available in the immediate surroundings of the sites. Freehand, hard-hammer percussion is the dominant flaking technique, although in some sites bipolar technique is also evident. Cores were reduced through simple flaking schemes, due either to difficulties in flaking low-quality chert, and/or because the short distance to raw material sources [98] were not conducive to longer reduction sequences. These patterns are maintained throughout time, with the only diachronic change being the appearance of morphologically discrete tool types in post- 1.3 Ma assemblages.

### Feiliang in the context of Out of Africa I

The Feiliang assemblage adds a new case study to the record of Mode 1 technologies in Eurasia >1 ma, currently interpreted within the context of Out of Africa I [3]. Early assemblages in Dmanisi [103], Atapuerca [104], Orce [105], Flores [106] and Nihewan (see this paper and references therein), among others, are characterized by a core and flake technology in which handaxes indicative of an Acheulean affinity are yet to be found. Given the early age of Dmanisi, the dominant hypothesis that Oldowan *Homo erectus* left Africa before the Acheulean emerged is currently the most plausible (but see Dennell [107] for alternative views), and would explain that Mode 1 assemblages are found across an enormous area from Southwestern Europe to Northeast Asia.

However, recognition of similarities on the main technological features of these assemblages should not overlook probable chronological and regional variability. In addition to differences in flaking schemes (e.g., variations in freehand and bipolar schemes; see a recent discussion in Yang et al., [28]), relative frequencies and characteristics of small retouched tools seem to vary widely across Eurasian assemblages. A more in-depth analysis of such variations, in addition to a systematic assessment of flaking methods, should be a priority of technological studies in forthcoming years; this shall lead to a better understanding of the earliest core-and-flake sites out of Africa, and to a more precise evaluation of inter-assemblage variability within a record that is currently all considered within one homogeneous, single label.

## Conclusions

The success of human migrations from Africa into the Nihewan Basin during the early Pleistocene was rooted on a suite of morphological and behavioral adaptations to new environments [13, 31, 107, 108]. Despite progress in the study of the Nihewan Early Pleistocene, several important issues still need to be addressed with regards to the nature of the archaeological sequence, typo-technological features, and adaptive behaviors of early hominin settlement of the basin. The current paper aims to contribute to such questions by presenting a systematic account of four relevant assemblages from the FL sequence, with particular emphasis on the archaeological sequence, integrity of the lithic assemblage, and technological behaviors adopted by the stone tool-makers. Several tentative conclusions can be drawn from our study:

The chronology of the FL site, dated to 1.2 Ma by paleomagnetism [33] suggests successive occupations of early hominins in the area from 1.66 Ma onwards [11], and contributes to making the Nihewan basin one of the most important areas for investigation of the archaeology of human origins during the ‘Out of Africa I’ [9, 11, 107–109].

The available evidence points to a primary depositional context of the FL archaeological assemblages. Our results indicate that relatively low densities of archaeological materials accumulated successively, and were buried rapidly in fine-grained sediments by gentle sheet wash events in a lake-margin environment.

The FL assemblage contains fossils of several mammal species, and some bones show fresh fractures that could evidence human action over some animals represented at the site. The lithic assemblage is typical of an Oldowan-like, Mode 1, core-and-flake technology. Like other Old World Mode 1 assemblages, the FL stone industry is characterized by a simple technological design, low degree of standardization, expedient flaking, and a few poorly standardized retouched flakes. Overall, cores indicate relatively simple flaking methods, with no clear organization and irregular use of any available flaking angles.

Extensive fieldwork at the Nihewan basin has produced a rich archaeological record, which now requires comprehensive and integrated studies of the taphonomic, technological and zooarchaeological aspects of each site. The application of standardized analytical methods will enable more systematic comparisons of inter-assemblage variability and, and as one of the oldest and densest concentrations of early Paleolithic sites in the world, the Nihewan Early Pleistocene archaeology should thus become a point of reference for reconstructions of early human behavior.

## Supporting information

S1 Appendix The Nihewan basin background, archaeological sequence and lithic analysis of FL. (PDF)

S1 Table FL stratigraphic sequences and grain size analysis of Trench TOK. (PDF)

S2 Table Primary dataset used for this paper. (XLSX)
