# Supplementary material for: Early Pleistocene archaeological occurrences at the Feiliang site, and the archaeology of human origins in the Nihewan Basin, North China
Source: PLoS One. 2017 Nov 22;12(11):e0187251. doi: 10.1371/journal.pone.0187251 (PMC5699830; doi:10.1371/journal.pone.0187251)
Supplement: S1 Appendix — (PDF) [file pone.0187251.s001.pdf]

FL Paper-Supporting Information

S1 Appendix. The Nihewan basin background, archaeological sequence and lithic analysis of FL.

1. The Nihewan basin

The Nihewan Basin, in Hebei Province ca. 150 km west of Beijing, is located in the transition zone between the North China Plain and the Inner Mongolian Plateau (Fig 1-a and b), and is filled with Pliocene to Holocene lacustrine and fluvial deposits [1-3]. The basin is about 200 km long and 15-30 km wide. At present, the Nihewan basin is characterized by a moderate semi-arid climate, with a mean annual temperature of ~ 7.7^{o} C and a mean annual rainfall of ~ 364 mm. Under the influence of the East Asian winter monsoon, winters are long and dry, with sub-freezing temperatures, while under the influence of the East Asian summer monsoon, summers are warm and humid [4, 5]. Today, the modern landscape is largely erosional, where extensive Pleistocene continental deposits are cut down by the modern drainage system of the Sanggan River and its tributary Huliu River (Fig 1c).

The Nihewan Basin is an inter-montane down-faulted basin at the northeastern margin of the Chinese Loess Plateau (Fig 1-a and b). Late Pliocene to middle-late Pleistocene fluvio-lacustrine sediments were widely deposited in the basin [3, 6-9]. The Nihewan Formation [10] represents the type section of the Early Pleistocene in North China [6], and is restricted to the lower part of the Nihewan Beds. Thick and continuous exposures of these fluvio-lacustrine sequences (without obvious tilting) are found mainly along the SW-NE trending Sanggan River and SE-NW trending Huliu River on the Cenjiawan Platform [11] to the eastern margin of the Nihewan basin, covering an area of some 20 km^2, and with a local elevation of >120 m (Fig 1-b and c).

Research in the Nihewan Basin was started by paleontologist Emile Licent in 1921,

when he initiated a search for fossils close to Nihewan village, followed by discovery of fossils at Xiashagou [12]. Further discoveries of vertebrate and invertebrate fossils were made by Licent, Barbour and Teilhard de Chardin between 1924 and 1929 [11, 13-16]. This led to the denomination of the Nihewan Fauna (also named Xiaoshagou Fauna), which was correlated to the Villafranchian fauna in Europe [11, 14, 15, 17], and has recently been magnetostratigraphically dated at ca. 2.2-1.7 Ma [9]. This fluvio-lacustrine sequence was named the Nihewan Beds [13] and, in the past several decades, a series of sedimentological, paleogeographical, geochemical, paleontological and palynological studies [7, 17-22] have contributed significantly to our understanding of the complex stratigraphy and depositional systems in the Nihewan Basin.

Archaeological interests in the Nihewan area were initially motivated by Licent and Teilhard de Chardin's discovery of a facetted stone which the Abbe Breuil [23] thought showed human modification, but was later deemed to be a natural specimen [24]. It was not until 1972 that undisputed evidence of hominin activity was identified in the form of a lithic artifact from Shangshazui [25, 26], a find that ultimately led to the discovery of Xiaochangliang (XCL) in 1978 [27], the first Early Pleistocene archaeological site recognized in the Nihewan basin. Since then, more than forty Early Pleistocene Paleolithic sites have been discovered in the Cenjiawan (Cheng-Chia-Wan) Platform in the eastern margin of the Nihewan basin (Fig 1c) [28-33], making Nihewan the area with the densest concentration of early Pleistocene Paleolithic sites outside Africa. The age of these Paleolithic sites has long been controversial due to the absence of suitable material for isotopic dating. In recent years, however, considerable progress has been made towards the paleomagnetic dating and excavation of several Nihewan sites, including Majuangou (MJG) and Xiaochangliang (XCL), among others.

2. The FL archaeological sequence

2.1 Trench 1

Trench 1 is at 934-935 m a.s.l. on the southwest slope of the Feiliang ridge at N 40°13′27.2", E 114°40′04.4". Located at c. 250 m northwest of Trench TOK, it was the first trench to be test excavated in 1986. Excavation in 1990 exposed an area of 17 m^2. The stone tool density at the trench is 6.53 per square meter. Ten stratigraphic layers of fine sand, sandy silt, silt, and clay were identified, spanning a total thickness of over 10 meters (Fig 1e, S1 Table 1). This was the thickest section exposed at the FL outcrop. The top of the section (layer 1) corresponds to the TBSU, layers 2 to 7 are correlated with the LU, and the lower part (layers 9 to 10) is associated with the BU of the Feiliang type section. Archaeological remains are limited to the middle and lower part of layer 3 and layer 4 (total thickness of one meter), and located five meters above the basal gravel layer.

Twenty-two lithic artifacts were collected from the section before excavation was conducted; subsequent excavation of 15 spits yielded 117 lithic artifacts (21.4% of the archaeological material recovered from the trench) and 431 fossil fragments (78.6%). Density of materials is higher in the north-northwest area of the trench (Fig 3-A), and is concentrated in 0.5 m of thickness. There is no sterile gap between the 15 spits, suggesting the existence of a single archaeological unit.

2.2 Trench 2

Trench 2 (N 40°13′27.5", E 114°40′03.5") is at 934-935 m a.s.l. on the southwest slope of the Feiliang ridge, c. 300 m to the northwest of Trench TOK. Excavations in 1996 exposed an area of 18 m^2 and six stratigraphic layers with a total thickness of over 3.5 m (Fig 1e, S1 Table 2), corresponding entirely to the LU of the type section. Archaeological remains are limited to sedimentological layers 3, 4, and the upper part of layer 5 (all composed of fine silts and clays), with a total thickness of 1.6 m. Twenty-nine spits were excavated in Trench 2, from which only 77 lithic artifacts were recovered, yielding the lowest stone tool density

(4.28/m^2) of the FL site complex. Fossil fragments (N=567) comprise 88.0% of the materials unearthed at the trench. Fig 3-B shows dispersion of materials through a depth of 1.6 m, with no apparent separation between them.

2.3 Trench 3

Trench 3 (N 40°13′26.3", E 114°40′05.7") is at 934-936 m a.s.l. on the southwest slope of the Feiliang ridge, c. 80 m to the northwest of Trench TOK. Excavations in 1996 exposed an area of 12 m^2 and 6 stratigraphic layers, with a total thickness of over 3 m (Fig 1e, S1 Table 3). This is the shortest section exposed at the FL site complex, and is attributed to the LU in the type section. Thirty-five spits were excavated, but archaeological remains were found only in layers 3, 4, and 5, in 1.95 m of fine silts and clays. Four lithic artifacts were collected prior to excavation of the trench, and only 92 lithic artifacts were recovered (7.67 artifacts/sq m) during excavation. In contrast, fossil fragments are abundant (N=518) and comprise 84.9% of the materials retrieved from the trench. As in Trench 2, archaeological materials are scattered with relatively low density through all excavated pits (Fig 3-C). Due to the lack of sterile levels across the 35 excavated spits, all artifacts were combined for analysis.

2.4 Trench TOK

Trench TOK (40°13′26.0", E 114°40′07.1", 935-937 m a.s.l.) is located to the southeast of the outcrop. It is the most southeastern and the largest trench of the FL site complex. Excavations in 1996 exposed over 35 m^2 and 13 stratigraphic layers with a total thickness more than 7 m (Fig 1e, S1 Table 4). The upper part (layer 1) can be correlated with the TBSU in the type section, while the remaining layers of the profile correspond to the LU. Eighteen spits were dug during excavations, and archaeological remains are limited to the three layers of silty clay (layers 9 to 11), having a total thickness of 2.10 m. With two surface and 669 *in situ* lithic artifacts (43.4% of the assemblage), Trench TOK has the highest stone artifact density (19.11/m^2) of all trenches, and contains a large number of fossils (N=871, 56.6% of the entire

assemblage). The density of materials is not homogeneous across the trench, with clusters of materials in the northeast versus lower density peaks in the southwest (Fig 3-D). Archaeological materials are evenly distributed across the 2 m stratigraphy of silty clays, with no sterile layers that could indicate separate depositional events.

3. The Lithic assemblages

3.1. Trench T1

T1 artifacts are predominantly of chert and brecciated chert (n=89, 64.5%), followed by siliceous dolomite (n=43, 31.1%) and other rocks (4.4%) (Fig 4-E).

Width/length (W/L) and thickness/breadth (Th/W) ratios (Fig 4-D) indicate that T1 flakes are primarily moderately short and relatively thick. Flake length averages 32.1 mm (range, 11 mm - 57 mm, s.d.=11.5) (Table 4), with most flakes clustering in the 2 to 4 cm size range (Fig 11-A). Flake striking platforms are normally cortical (68.9%) or show one scar (28.9%), while only 2.2% have two or more scars (Fig 11-B). Following Toth's categories (Fig 11-C), there is a high proportion of type V (40.0%) and VI (31.1%) flakes (Fig 11-D). Flakes with non-cortical dorsal surfaces dominate the assemblage (34.6%) (Fig 11-E). Flake scar patterning is most commonly unidirectional (55.6%), followed by bidirectional and transverse patterns (see percentages in Fig 11-F). The platform angle of T1 flake butts is obtuse and relatively variable, varying between 80° and 130° (mean=105.6°, s. d.=11.9°). Some selected flakes are shown in Fig 12-A.

Cores (N=8) represent 6.1% of the assemblage, unmodified material excluded (Fig. 4-C). Core scrapers (n=4) predominate in core categories (Table 5). Core dimensions range between 57 and 102 mm (mean=75.6mm, s.d.=13.1) (Fig 8-A), and weight ranges between 106 and 731 grams (mean= 381.9, s. d.=197.7) (Table 4). Most cores were made from blocks and cobbles probably available in the immediate surrounding, and they usually preserve large areas of cortex on their surfaces (20 - 90%, mean=63.8%, s.d.=23.9) (Fig 8-C and D). Half

the T1 cores have more than 6 negative scars, with an average of 8 (Fig 8-B). Core edge angle ranges between 80° and 97° (s.d.=5.73), with a mean of 83.4°. It is presumed that cores were abandoned when flaking was still feasible.

As shown in Table 6, 75.0% (n=6) of cores are unifacially flaked. With regards to knapping methods, a bifacial abrupt partial (BAP) strategy predominates, with varying proportions of other flaking schemes (see counts in Table 7) (see Fig 9, a-d).

Five, relatively small retouched flakes (3.8% of the assemblage) were identified, with length ranging from 22 to 29 mm (mean =26.0 mm, and s.d.=3.1), and weighing from 9 to 15 grams (mean = 12.4 grams, s.d.=2.6) (Table 4). Retouch, which is non-systematic, casual and usually direct (i.e. on the dorsal surface), was applied to the lateral edge of whole flakes (Fig 13-A).

3.2. The T2 assemblage

T2 artifacts are primarily of chert and brecciated chert (n=52, 67.5%), followed by siliceous dolomite (n=14, 18.2%). The remaining artifacts (11.7%) are of other rocks including quartz/quartzite, and granite, with only 2.6% on lava (Fig 4-E).

Width/length (W/L) and thickness/breadth (Th/W) ratios (Fig 4-D) indicate that T2 flakes are moderately thin and relatively short. Flakes average 35.2 mm in length (Table 4 and Fig 11-A), flake striking platforms are normally unifacetted (50.0%) or cortical (28.6%), and 21.4% have two or more scars (Fig 11-B). There are high proportions of Toth's type V (35.7%) and VI (35.7%) flakes (Fig 11-C), followed by types II (21.4%) and III (7.2%). Overall, 42.9% of flakes bear no cortex on their dorsal surfaces, and have an average of 3.9 previous scars (Fig 11-D). Aside from flakes with non-cortical dorsal surfaces (50.0%), cortex is mostly located on the left (28.6%) and right (21.4%) areas of flakes (Fig 8-E). Flake scar patterning tends to be unifacial (57.1%; Fig 11-F), followed by transverse and bidirectional patterns (28.6% and 14.3%, respectively). The platform angle is obtuse and

ranges between 94° and 124° (mean=108.5°, s.d.=9.5°) (see selected flakes in Fig 11-B).

Cores (N=6) represent 7.9% of the assemblage, unmodified material excluded (Fig 4-C), with dimensions ranging between 42 and 91 mm (mean=68.2mm, s.d.=18.9) (Fig 8-A), and weighing between 34 and 567 grams (mean=266.0, s.d.=186.3) (Table 4).Typologically, polyhedrons and test cores (n=2 each) predominate (Table 5). As with T1, most cores were made from blocks and cobbles, and usually preserve cortical surfaces (ranging from 15 % to 75%, mean=45%, s.d.=26.5) (Fig 8, C and D). As shown in Fig. 8-B, 66.7% of T2 cores have more than 6 scars, with an average of 7.2 scars. Core edge angles vary between 73° to 92° (s.d.=7.8°), with a mean of 82.7°.

Half (n=3) the cores are multifacially flaked (multi-directional), 33.3% (n=2) are unifacially flaked (unidirectional), and one core is bifacially flaked (bidirectional) (Table 6). With regards to knapping methods, UAP and Multifacial (n=2 each) predominate (see Table 7 and Fig 9, e-f).

The two retouched flakes (2.6% of the assemblage) identified have considerably different length (31mm and 81mm) and weight (14 grams and 289 grams respectively) (Table 4). Both retouched flakes were on flake fragments, and retouch, which is unsystematic and casual, affects the lateral edge of the blank.

3.3. The T3 assemblage

T3 artifacts are primarily of chert and brecciated chert (n=74, 77.1%), followed by siliceous dolomite (n=14, 14.6%). 7.3% are on other rocks including quartz/quartzite and granite, and only 1.0% are of lava (Fig 4-E).

Width/length (W/L) and thickness/breadth (Th/W) ratios (Fig. 4-D) indicate that flakes are primarily short and relatively thick. Flake length is 27.8 mm (ranges from 10mm to 88 mm, s.d.=17.2) (Table 4, Fig 11-A). Striking platforms are mostly cortical (65.2%) and unifacetted (26.1%) (Fig 8-B). Toth’s types VI (56.5%) and V (30.4%) predominate (Fig 11-

C). 56.5% of the flakes have non-cortical dorsal surfaces, and have an average of 4.1 previous scars (Fig 11-D). Flakes with non-cortical dorsal surfaces dominate (56.5%), followed by equal percentages (8.7%) of flakes with cortex on their left, right and distal sides (Fig 11-E). Scar patterning indicates a dominance of unidirectional flaking (60.9%) (Fig 11-F), followed by transverse and bidirectional flaking (both 17.4%). T3 flakes have obtuse and slightly variable platform angles, varying between 89° and 116° (mean=102.9°, s. d.=7.1°) (see examples in Fig 12-C).

Cores (N=6) represent 6.8% of the assemblage, unmodified material excluded (Fig 4-C), with dimensions ranging between 27 and 78 mm (mean=46.2mm, s.d.=19.2) (Fig 8-A), and weighing between 11- 661 grams (mean=145.2, s. d.=254.1) (Table 4). Typologically, T3 cores are classified as core scrapers (n=3), polyhedrons (n=2), and one test core (Table 5). Three cores are on blocks, two are on fragments, and one is on a cobble. The percentage of surface cortex is usually low, ranging from 5% to 85% (mean=30%, s.d.=28.6) (Fig 8, C and D). Fig.8-B shows an average of 6.3 scars per core. Core edge angles vary from 71° to 95° (s.d.=9.7°), with a mean of 80.5°.

As shown in Table 6, 50% (n=3) of the cores are multifacially flaked (multi-directional), 33.3% (n=2) are unifacially flaked (unidirectional), and one is bifacially flaked (bidirectional). There is predominance of UAP and Multifacial (n=2 each) knapping methods (Table 7 and Fig, 9, g-i)

One retouched tool (1.5%) was identified; it is small (26 mm long), weighs 7 grams, was made on a whole flake, and its lateral and distal edges show non-systematic retouch.

3.4. The TOK assemblage

Chert and brecciated chert (n=584, 87.1%) dominate the TOK lithic assemblage (Fig 4-E), followed by 8.1% siliceous dolomite (n=54), and 4.8% other raw materials.

Width/length (W/L) and thickness/breadth (Th/W) ratios (Fig 4-D) indicate that TOK

flakes are moderately short and relatively thick (Fig 12-D). Flakes average 35.3 mm in length (range from 7 mm to 86 mm, s.d.=13.1) (Table 4, Fig 11-A). Flake striking platforms are mostly cortical (43.0%) or have one scar (36.5%) (Fig 11-B). There are high proportions of Toth's type VI (34.6%) and V (29.9%) flakes (Fig 11-C). 47.7% of flakes have no dorsal surface cortex, and have an average of 3.4 previous scars (Fig 11-D). Analysis of cortex on dorsal surfaces shows dominance of non-cortical patterns (47.7%) (Fig 11-E). Unidirectional flake scar patterning is most common (54.2%) (Fig 11-F), followed by transverse (20.5%) and bidirectional patterns (19.6%) respectively. Platform angles are obtuse, varying between 82° and 127° (mean=106.6°, s.d.=10.1°).

Cores (N=30) make up 4.8% of the sample (Fig 4-C), unmodified material excluded. Typologically, TOK cores can be classified as polyhedrons (n=11), core scrapers (n=6), bifacial discoids (n=5), test cores (n=5), bifacial choppers (n=2), and a unifacial chopper (n=1) (Table 5). Maximum dimensions range between 32 and 156 mm (mean=79.7mm, s.d.=32.9) (Fig 8-A), and between 19 and 2479 grams (mean= 489.9 grams, s.d.=645.7) (Table 4). Most cores were made from blocks (40.0%), angular fragments (33.3%), and cobbles (26.7%) (Fig 8-D), and usually preserve small areas of cortex on their surfaces (0 % to 85%, mean=32.7%, s.d.=27.4) (Fig 8-C). A substantial number (50%) have more than 9 scars (Fig 8-B), and the average is 8.8 scars per core. Core edge angles range from 69° to 98° (s.d.=7.7°), with a mean of 81.1°.

As shown in Table 6, 40.0% (n=12) of cores are multifacially flaked, 36.7% (n=11) are bifacially flaked (bidirectional), and 23.3% (n=7) show unifacial flaking. Multifacial flaking methods are more common (40%, n=12), followed by bifacial peripheral (BP) (16.7%, n=5), UAP (13.3%, n=4), and others (Table 7, Fig 10).

Fourteen retouched flakes (2.2% of the assemblage) were identified (Fig 4-C). Overall, retouched pieces are relatively small (length ranges from 19 to 45 mm, mean= 32.9 mm,

s.d.=8.1), and weight ranges from 6 to 47 grams (mean=18.9 grams, s.d.=12.1) (Table 4). Whole flakes (n=10) and flake fragments (n=4) were chosen for retouch. Eleven pieces show direct retouch, two inverse retouch (i.e., on the ventral surface), and one bears bifacial retouch. Retouch is commonly located on the lateral edge (n=10) of blanks, although 3 pieces are retouched on both lateral and distal edges, and one artifact is retouched on the distal edge. Six retouched flakes show denticulate retouch, five pieces have rectilinear retouch, and two show convex and concave retouch. Retouched angles range from 55° to 77° (s.d.=7.1°), with a mean angle of 66.2°. In general, retouch is unsystematic and casual (Fig 13-B).

References

1. Zhu RX, Hoffman KA, Potts R, Deng CL, Pan YX, Guo B, et al. Earliest presence of humans in northeast Asia. Nature. 2001; 413: 413-417.
2. Zhu R X, Potts R, Xie F, Hoffman KA, Deng CL, Shi CD, et al. New evidence on the earliest human presence at high northern latitudes in Northeast Asia. Nature. 2004; 431: 559-562.
3. Deng CL, Zhu RX, Zhang R, Ao H, Pan YX. Timing of the Nihewan formation and faunas. Quatern Res. 2008; 69: 77-90.
4. Dennell R W. The Nihewan basin of North China in the Early Pleistocene: continuous and flourishing, or discontinuous, infrequent and ephemeral occupation? Quatern Int. 2013; 295: 223-236.
5. Ao H, Dekkers MJ. An ZS, Xiao GQ, Li YX, Zhao H, et al. Magnetostratigraphic evidence of a mid-Pliocene onset of the Nihewan Formation implications for early fauna and hominid occupation in the Nihewan Basin, North China. Quatern Sci Rev. 2013; 59: 30-42.
6. Young CC. The Plio-Pleistocene boundary in China. In: Report on 18th International Geological Congress, London. 1950. 115-125.
7. Zhou TR, Li H Z, Liu QS, Li RQ, Sun XP. Study on the Cenozoic paleogeograpgy of Nihewan Basin. Beijing: Science Press, 1991. pp. 1-162.
8. Zhu RX, An ZS, Potts R, Hoffman KA. 2003. Magnetostratigraphic dating of early humans in China. Earth-Sci Rev, 2003; 61: 341-359.

9. Liu P, Deng CL, Li SH, Cai SH, Cheng HJ, Yuan BY, et al. Magnetostratigraphic dating of the Xiashagou Fauna and implication for sequencing the mammalian faunas in the Nihewan Basin, North China. Palaeogeogr Palaeoclimatol Palaeoecol. 2012; 315-316: 75-85.

10. Min LR, Chi ZQ. Quaternary Geology of the Western Yangyuan Basin (in Chinese with English abstract). Geological Publishing House, Beijing. 2003.

11. Barbour GB, Licent E, Teilhard de Chardin P. Geological study of the deposits of the Sangkanho basin. Bull Geol Soc Chin. 1927; 5: 263-278.

12. Licent E. Voyage aux terrasses du Sangkan Ho. Publications du Muse´e Hoang Ho Pai Ho (Tien Tsinn) 1924. 4: 1–14.

13. Barbour GB. Preliminary observation in the Kalgan Area. Bulletin of Geological Society of China. 1924; 3: 153-168.

14. Barbour GB. The deposits of the Sangkanho Valley. Bull Geol Soc Chin. 1925; 4: 53-55.

15. Teilhard de Chardin P, Piveteau J. Les mammiferes fossils de Nihowan (Chine). Ann de Paleontol. 1930; 19: 1-154.

16. Keates SG. Early and Middle Pleistocene Hominid Behavior in Northern China. BAR International Series 863. John and Hedges, Oxford, 2000. pp. 1-107.

17. Qiu ZX. Nihewan fauna and Q/N boundary in China. Quatern Sci. 2000; 20: 142-154.

18. Chen MN. 1988. Study on the Nihewan Beds. Beijing: Ocean Press. 1988. 1-145.

19. Tang YJ, LiY, ChenWY. Mammalian fossil and the age of Xiaochangliang Paleolithic site of Yangyuan, Hebei. Vert PalAsiat. 1995; 33: 74-83.

20. Yuan BY, Zhu RX, Tian WL, Cui JX, Li RQ, Wang Q, et al. 1996. Magnetostratigraphic dating on the Nihewan Formation. Sci in China (Ser D)1996; 26: 67-73.

21. Pei SW, Li XL, Liu DC, Ma N, Peng F. Preliminary study on the living environment of hominids at the Donggutuo site, Nihewan Basin. Chin Sci Bull. 2009: 54(21): 3896-3904.

22. Li XL, Pei SW, Jia ZX, Guan Y, Niu DW, Ao H. Paleoenvironmental conditions at Madigou (MDG), a newly discovered Early Paleolithic site in the Nihewan Basin, North China. Quatern Int. 2016; 400: 100-110.

23. Breuil H. L’etat actuel de nos connaissances sur les industries paleolithiques de Choukoutien. Anthropologie. 1935; 45: 745-746.

24. Movius HL. The Lower Palaeolithic cultures of southern and eastern Asia. Trans Am Phil Soc. 1948; 38(4): 329-420.
25. Gai P, Wei Q. Discovery of a stone artifact from Lower Pleistocene, Nihowan. Vertebra Pal. 1974; 12: 70-72.
26. Wei Q, Pei SW, Feng XW, Ao H, Jia ZX. The stone artifacts from Shangshazui, Nihewan Basin. Acta Anthropol Sin. 2015; 34(2):139-148.
27. You Y, Tang YJ, Li Y. 1980. Paleolithic discoveries in the Nihewan formations. Chinese Quatern Res. 1980; 5: 1-13.
28. Wei Q, Xie F. Selected treatises on Nihewan. Beijing: Cultural relics Publishing House. 1989.
29. Xie F. Nihewan. Cultural Relics Publishing House, Beijing. 2006.
30. Xie F, Li J, Liu LQ. Paleolithic archeology in the Nihewan Basin. Shijiazhuang: Huashan Literature &Arts Press. 2006. pp. 1-278.
31. Wei Q, Li J, Pei SW. Paleolithic sites and ancient hominids culture. In: Yuan BY, Xia ZK, & Niu PS, eds, Nihewan Rift and Paleoanthropology. Beijing: Geological Publishing House. 2011. pp. 132-207.
32. Pei SW, Ma N, Li XL. A report on the discovery of some Paleolithic localities on the eastern part of Nihewan Basin in 2007. Acta Anthropol Sin. 2010; 29(1): 33-43.
33. Yang SX, Hou YM, Yue JP, Petraglia MD, Deng CL, et al. The lithic assemblages of Xiaochangliang, Nihewan basin: Implications for Early Pleistocene hominin behaviour in North China. PLoS ONE. 2016;11(5): e0155793. doi:10.1371/journal. pone.0155793.
