# Supplementary material for: Early Pleistocene archaeological occurrences at the Feiliang site, and the archaeology of human origins in the Nihewan Basin, North China
Source: PLoS One. 2017 Nov 22;12(11):e0187251. doi: 10.1371/journal.pone.0187251 (PMC5699830; doi:10.1371/journal.pone.0187251)
Supplement: S1 Table — (PDF) [file pone.0187251.s002.pdf]

FL Paper-Supporting Information

S1 Table. FL stratigraphic sequences and grain size analysis of Trench TOK.

S1 Table 1. Stratigraphic sequence of Trench 1.

Layer	Stratigraphic description	Thickness (m)	Lithics	Fossils
1(top)	Grayish black silt and fine sand, loose structure	0.30	Absent	Absent
2	Yellowish brown sandy clay with two calcareous layers	1.74	Absent	Absent
3	Grayish green clay with a low content of calcareous concretions	0.80	Present (middle and lower part only)	Present (middle and lower part only)
4	Grayish yellow fine silt, horizontal bedding	0.20	Present	Present
5	Brownish green clay with a high content of yellow oxidation spots and calcareous concretions	0.90	Absent	Absent
6	Yellowish green clay, horizontal bedding	0.55	Absent	Absent
7	Grayish black clay,	0.35	Absent	Absent
8	Yellowish red sandy clay	2.30	Absent	Absent
9	Grayish green clay sand with variable sized small pebbles and breccia, cross-bedding in the lower part	1.10	Absent	Absent
10 (bottom)	Gravel layer interbedded with yellowish green coarse sand	>1.80	Absent	Absent

S1 Table 2. Stratigraphic sequence of Trench 2.

Layer	Stratigraphic description	Thickness (m)	Lithics	Fossils
1(top)	Light yellow fine sand interbedded with silt, loose structure, with small calcareous concretions	1.00 -1.30	Absent	Absent
2	Grayish yellow sandy clay with calcareous layers , cross bedding in lower part	0.00 -0.55	Absent	Absent
3	Grayish green clay with two gray calcareous layers , horizontal bedding in lower part	1.20 -0.80	Present	Present
4	Brownish yellow fine silt with a high content of yellow oxidation spots, cross bedding in lower part	0.40 -0.60	Present	Present
5	Gray and grayish black clay interbedded with sandy nodules, and a high content of calcareous concretions	0.30	Present (upper part only)	Present (upper part only)
6 (bottom)	Gray and grayish green clay	>0.5	Absent	Absent

S1 Table 3. Stratigraphic sequence of Trench 3.

Layer	Stratigraphic description	Thickness (m)	Lithics	Fossils
1(top)	Yellowish brown clay	0.10-0.20	Absent	Absent
2	Light yellow fine sand interbedded with silt, loose structure, with two layers of calcareous concretions and nodules	0.60	Absent	Absent
3	Grayish green clay with two calcareous layers in the middle area and calcareous concretions in lower part, horizontal bedding in the upper part	0.90	Present	Present (upper part only)
4	Grayish yellow fine silt interbedded with clay nodules in the lower part, with a high content of yellow rust spots and ferriferous nodules, and a high content of shell fragments	0.62	Present	Present
5	Grayish yellow silty clay with a high content of yellow oxidation spots and calcareous concretions, with a calcareous layer in the bottom, and a low content of shell fragments	0.90	Present (upper part only)	Present (upper part only)
6 (bottom)	Grayish black clay	>0.30	Absent	Absent

S1 Table 4. Stratigraphic sequence of Trench TOK.

Layer	Stratigraphic description	Thickness (m)	Lithics	Fossils
1(top)	Brown fine sand, loose structure (TBS beds)	0.95	Absent	Absent
2	Grayish silty clay with some calcareous concretions	0.70	Absent	Absent
3	Brownish red clay, compact structure	0.12	Absent	Absent
4	Yellow fine sand, ripple bedding	0.30	Absent	Absent
5	Brownish clay, ripple bedding in the lower part	0.55	Absent	Absent
6	Grayish green silty clay, ripple and cross bedding, with a high content of yellow oxidation spots	0.35-0.70	Absent	Absent
7	Brown silty clay, horizontal bedding in lower part, with some yellow oxidation spots	0.50-0.80	Absent	Absent
8	Grayish yellow fine sand, ripple bedding in lower part	0.30-0.35	Absent	Absent
9	Light brown and gray silty clay, with high content of calcareous concretions	0.90-1.40	Present	Present
10	Gray silty clay, with calcareous layers	0.20-0.90	Present	Present
11	Grayish yellow and grayish green clay	0.40-0.90	Present	Present
12	Light grayish silt and grayish green clay silt, cross bedding	0.20-0.60	Absent	Absent
13(bottom)	Brownish red and grayish green clay, horizontal bedding	>0.33	Absent	Absent

S1 Table 5. Grain size analysis of the FL section and archaeological layers at the TOK trench.

Size classes	Size range	TOK section	Archaeological layers
		Percent	Percent
granules and small pebbles	>0.25 mm	1.90	0.84
fine sand	0.25-0.125 mm	7.33	3.89
very fine sand and coarse silt	0.125-0.0156 mm	53.70	54.27
fine silt to clay	<0.0156 mm	37.07	41.00
